# Enhanced efficiency of virulent and temperate phage combination mediated through bacterial membrane vesicles

**DOI:** 10.1128/jvi.00941-25

**Published:** 2025-11-26

**Authors:** Panida Saeju, Ampapan Naknaen, Pongsakorn Sukonthamarn, Anchalee Tassanakajon, Poochit Nonejuie, Vorrapon Chaikeeratisak

**Affiliations:** 1 Department of Biochemistry, Faculty of Science, Chulalongkorn University 26683, Bangkok, Thailand; 2 Center of Excellence for Molecular Biology and Genomics of Shrimp, Department of Biochemistry, Faculty of Science, Chulalongkorn University 26683, Bangkok, Thailand; 3 Department of Biomedical Sciences and Biomedical Engineering, Faculty of Medicine, Prince of Songkla University 37689 , Songkhla, Thailand; 4 Center for Advanced Therapeutics, Institute of Molecular Biosciences, Mahidol University 98841, Nakhon Pathom, Thailand; Michigan State University, East Lansing, Michigan, USA

**Keywords:** *Vibrio parahaemolyticus*, bacteriophage, AHPND, temperate phages, phage cocktail, membrane vesicles

## Abstract

Since multidrug-resistant *Vibrio parahaemolyticus* (VP), a deadly pathogenic bacterium responsible for acute hepatopancreatic necrosis disease (AHPND), which has increasingly emerged, bacteriophages have been focused on as an alternative therapy against drug-resistant bacterial infections. Here, we identified various distinct vibriophages, as evidenced by host range assays and restriction fragment length polymorphism analysis. Since we aimed to enhance the efficiency of our previously developed vibriophage cocktail, Eric and Ariel (EA), which has been demonstrated to be effective against VP_AHPND_, each selected phage was combined with the EA cocktail to evaluate the efficiency of formulated cocktails against VP_AHPND_ isolates. The result revealed that supplementation of the EA cocktail with the phage PhiPS02 yielded outstanding outcomes, particularly against the VP_AHPND_ strain KT1001. The enhanced efficacy of this formulated cocktail was further validated *in vivo*, where it rescued infected shrimp through a significant reduction of bacterial toxins, leading to approximately 75% survival. PhiPS02 is a novel temperate vibriophage and harbors a genome of 34,737 base pairs encoding genes involved in the lysogenic life cycle. Under physiological stresses that induced prophage activation, PhiPS02-lysogenized bacteria exhibited membrane blebbing and produced small membrane vesicles (MVs) with distinct biomolecular constituents. Interestingly, the combination of lysogen-derived MVs with the EA phage cocktail substantially enhanced bacterial suppression, compared to that of MVs from wild-type bacteria, suggesting a synergistic interaction between lysogen-derived MVs and phages. This study highlights the complex interplay among phages and their bacterial hosts, mediated through lysogen-derived MVs, and provides a novel strategy for the application of temperate phages in the management of VP_AHPND_ in aquaculture.

**IMPORTANCE:**

Since the resurgence of phage therapy as a strategy to combat antibiotic-resistant bacterial infections, the use of combined virulent phages has shown promising therapeutic potential. However, naturally occurring phages are predominantly temperate, restricting their use as therapeutic agents due to concerns regarding their ability in lysogenic conversion. Here, we demonstrate that temperate phages can confer a therapeutic potential by inducing lysogenized bacteria to produce small membrane vesicles (MVs) that synergize with virulent phages in combination to suppress bacterial growth. Since MVs originate from the bacterial outer membrane, they not only retain phage receptors but also carry phage-derived biomolecules influenced by resident prophage. Potentially, they can mediate receptor transfer to phage-resistant strains via cell-MV fusion and improve phage infectivity through the combined action of phages and prophage-derived enzymes. Our findings provide insights into the role of lysogen-derived MVs and offer strategy into a novel strategy for harnessing temperate phages in therapeutic applications.

## INTRODUCTION

*Vibrio parahaemolyticus* (VP) is a Gram-negative, halophilic bacterium widely distributed in marine environments and aquaculture systems. It is recognized as a major foodborne pathogen responsible for human gastroenteritis, normally associated with the consumption of raw or undercooked seafood contaminated with VP (1). In addition to its impact on human health, VP poses a significant threat to marine life. Certain VP strains can cause acute hepatopancreatic necrosis disease (AHPND), which is one of the most devastating diseases in shrimp aquaculture. In particular, these AHPND-causing VP (VP_AHPND_) strains harbor a unique plasmid, pVA1, and thus are capable of encoding the PirAB^vp^ binary toxin genes (2). These toxins are causative agents of AHPND, in which pathogenicity is characterized by degeneration of the hepatopancreas, often turning pale to white in appearance, accompanied by lethargy, anorexia, slow growth, and an empty gastrointestinal tract. Mortality rates can approach nearly 100% within the first 35 days of the post-larval stage (3). Since its emergence, AHPND has rapidly spread across several shrimp-producing countries worldwide, resulting in massive shrimp mortality and subsequent substantial losses to the aquaculture industry (4).

Antibiotics have traditionally been employed to control and prevent VP infections in aquaculture (5). However, the extensive and improper use of antibiotics has accelerated the emergence of multidrug-resistant (MDR) vibrios, further contributing to the increasing incidence of treatment failures (6). Moreover, given the slow pace of novel and effective antibiotic discovery that has never caught up with the drug-resistant rate, alternative therapeutic strategies are urgently needed to manage bacterial infections in aquaculture settings. Bacteriophages or phages, which are prokaryotic viruses that specifically rely on bacterial hosts for reproduction, have garnered significant attention as a promising alternative according to their targeted antimicrobial function against bacteria (7, 8). To date, the idea of using phages as therapeutic agents against bacterial diseases has been revived, as evidenced by an increasing number of studies about phage therapy (9). Although the use of individual phages against a pathogen usually yields efficient outcomes, the use of multiple phages, known as a phage cocktail, is highly preferable (10). Phage cocktails composed of multiple phages can overcome several limitations associated with single-phage therapies, including the narrow host spectrum and rapid development of phage resistance. Through incorporating diverse phages, cocktails can broaden the host range, prolong bacterial suppression, and reduce the emergence of resistance (11), ultimately enhancing the overall therapeutic efficacy.

To formulate effective phage cocktails for therapy, lytic phages are generally preferred due to the obligate lysis of bacterial cells at the end of their replication cycle. In contrast, temperate phages are typically avoided due to concerns regarding their intrinsic nature to enter lysogeny and their potential for horizontal gene transfer, which would be harmful and may compromise treatment efficacy (12, 13). However, the isolation of lytic phages targeting particular bacterial species, such as *Clostridium difficile* (14), *Burkholderia cepacia* complex (15), and *Mycobacteria* (16), remains challenging. This difficulty is partly attributed to the predominance of temperate phages within natural phage populations, which arises from the co-evolution between phages and their bacterial host. Through a lysogenic relationship, whereby temperate phages integrate their genome into the host chromosome, they gain evolutionary advantages that ensure the long-term stability of the phage genome within bacterial populations over successive generations (12, 15). Given these challenges, there has been increasing interest in exploring possible strategies to harness temperate phages for therapeutic purposes. Current approaches under investigation include the use of temperate phages to attenuate bacterial virulence via lysogenic conversion (17, 18) and the development of temperate phage-based cocktails (14, 15).

Even though certain combinations of lytic and temperate phages have demonstrated promising therapeutic potential, the mechanisms underlying their synergistic interactions remain poorly understood. There has been growing evidence demonstrating that bacterial membrane vesicles (MVs), typically produced across bacterial species, play multifaceted roles in modulating phage-bacterium interactions (19, 20). Beyond their common functions as a unique bacterial secretion pathway involved in horizontal gene transfer and the delivery of biomolecular cargo to neighboring bacteria (21, 22), MVs can also influence phage activity in diverse ways, with unpredictable outcomes being dependent on several factors, such as the bacterial species producing the vesicles. For instance, they can facilitate phage infectivity via receptor transfer (23) or act as decoys that interfere with phage infections (24). More interestingly, recent studies have demonstrated that, not only being produced by bacteria themselves, MVs can also be generated through prophage induction in certain lysogens, resulting in vesicles with unique structural and compositional characteristics (25–28). These findings raise the possibility that certain temperate phages may participate in cellular signaling to regulate phage-host interplay through MV-mediated pathways.

Here, we identified a novel temperate vibriophage, PhiPS02, which exhibited enhanced bacterial suppression against virulent VP_AHPND_ strains when combined with two previously characterized lytic vibriophages, Eric and Ariel (EA) (29). The efficacy of this phage combination was validated through both *in vitro* experiments and an *in vivo* infection model, in which it demonstrated potent activities in bacterial suppression and significant reduction of bacterial toxin, subsequently leading to the increased shrimp survival rates during VP_AHPND_ infection. Notably, the combination of small-sized MVs with unique composition derived from PhiPS02-lysogenized strains to the EA cocktail substantially enhanced antibacterial activity, suggesting the mechanism by which the temperate vibriophage improves the overall potency of the phage combination through bacterial MVs and highlighting a role of temperate phages in shaping therapeutic outcomes via bacterial MVs.

## RESULTS

### Isolation and selection of vibriophages based on restriction fragment length polymorphism and host spectrum against VP_AHPND_ strains

**Fig 1:**
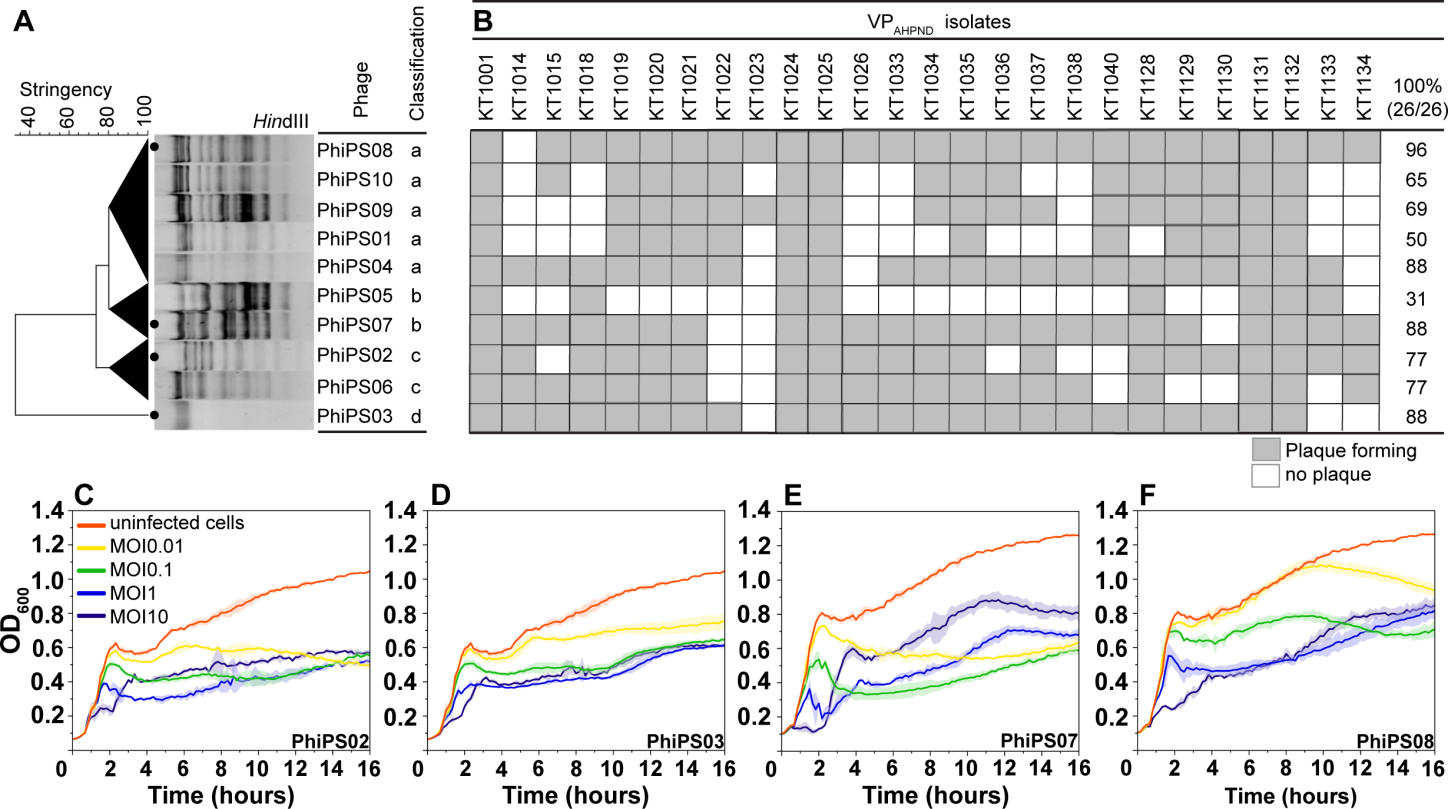
Clustering-based vibriophage selection and their killing kinetics. (**A**) Clustering based on patterns of RFLP of phage genomes digested with the enzyme *Hin*dIII by using the unweighted pair group method with arithmetic mean clustering technique. (**B**) Host spectrum analysis of 10 isolated vibriophages against 26 VP_AHPND_ strains. The gray and white boxes indicated visible plaques and no plaques, respectively. (**C–F**) Bacterial cell lysis profile of candidate phages, including (**C**) PhiPS02, (**D**) PhiPS03, (**E**) PhiPS07, and (**F**) PhiPS08, at different MOIs at 0.01, 0.1, 1, and 10 over a 16-hour period of treatment.

Rearing water was collected from aquatic farms to isolate and enrich phages targeting deadly VP_AHPND_ strains that are locally spreading in southern Thailand. Through the phage screening process, 10 phages were successfully isolated: PhiPS01, PhiPS02, PhiPS03, PhiPS04, PhiPS05, PhiPS06, PhiPS07, PhiPS08, PhiPS09, and PhiPS10 (Fig. 1A). To distinguish different phages and group closely related ones, we performed restriction fragment length polymorphism (RFLP) analysis of their DNA genomes. Upon treatment with different restriction enzymes, *Bam*HI and *Eco*RI did not digest any of the phage genomes (Fig. S1), indicating the absence of restriction sequences of these enzymes in the phage genomes. However, *Hin*dIII was capable of digesting most phage genomes, resulting in distinct digestion patterns for most phages, except for PhiPS03, whose genome was not recognized by the enzyme (Fig. 1; Fig. S1). Clustering analysis of the *Hin*dIII digestion pattern at 80% stringency further revealed that some phages are genetically closely related, thereby falling in the same group (Fig. 1A; dark triangle). This classification divided the phages into four main groups: Group a, including PhiPS01, PhiPS04, PhiPS08, PhiPS09, and PhiPS10; Group b, including PhiPS05 and PhiPS07; Group c, including PhiPS02 and PhiPS06; and Group d, consisting solely of PhiPS03. To further investigate the potential of these vibriophages in targeting VP_AHPND_ strains, we then evaluated the ability of phages to form plaques against 26 hosts to assess their host spectrum. The result revealed that the degree of host specificity against VP_AHPND_ strains varied among them, ranging from 31% to 96% (Fig. 1B). Interestingly, despite their genetic similarities and classification within the same groups (Groups a, b, and c), individual phages displayed distinct host spectra. These data suggest subtle phenotypic variations among closely related phages, confirming their difference despite being grouped.

### Efficiency of selected phages in suppressing the growth of VP_AHPND_ strains

We selected a representative from each group (Groups a, b, c, and d) based on the breadth of their host range against VP_AHPND_ strains (Fig. 1A and B). Among the phages in each group, we selected PhiPS02, PhiPS03, PhiPS07, and PhiPS08 (Fig. 1A; black dots) as promising candidates from Groups c, d, b, and a, respectively, as they exhibited the broadest host range, effectively targeting 77%, 88%, 88%, and 96% of the tested bacterial isolates. To evaluate the lytic activity of these four candidate phages against VP_AHPND_ at finer temporal resolution, we monitored bacterial cell density over a 16-hour treatment period at various multiplicities of infection (MOIs) (Fig. 1C through F). In the absence of phages, the bacterial cell density at OD_600_ increased continuously from approximately 0.06 to around 1.2 over 16 hours (Fig. 1C through F; red line). In contrast, phage-treated cultures exhibited lower bacterial densities at all tested MOIs, indicating that bacterial cell growth was suppressed when the phage was present in the culture. However, the degree and pattern of bacterial suppression varied in a dose-dependent manner. At low MOIs (0.01, 0.1, and 1), bacterial cell density initially increased over the first 2 hours in an inverse correlation with the MOIs (Fig. 1C through F; yellow, green, and blue lines), followed by a slight decrease and then remaining at comparable levels until the end of treatment regardless of the MOIs used. In contrast, at a high MOI of 10 (Fig. 1C through F; purple lines), bacterial growth was strongly suppressed within the first 2 hours but then gradually rebounded until the end of the experiment. These data confirmed that the selected phages PhiPS02, PhiPS03, PhiPS07, and PhiPS08 effectively inhibit the growth of VP_AHPND_, with suppression pattern variation depending on the MOI applied.

### The phage cocktail supplemented with PhiPS02 demonstrates potent activity against VP_AHPND_ in both *in vitro* and *in vivo* experiments

**Fig 2:**
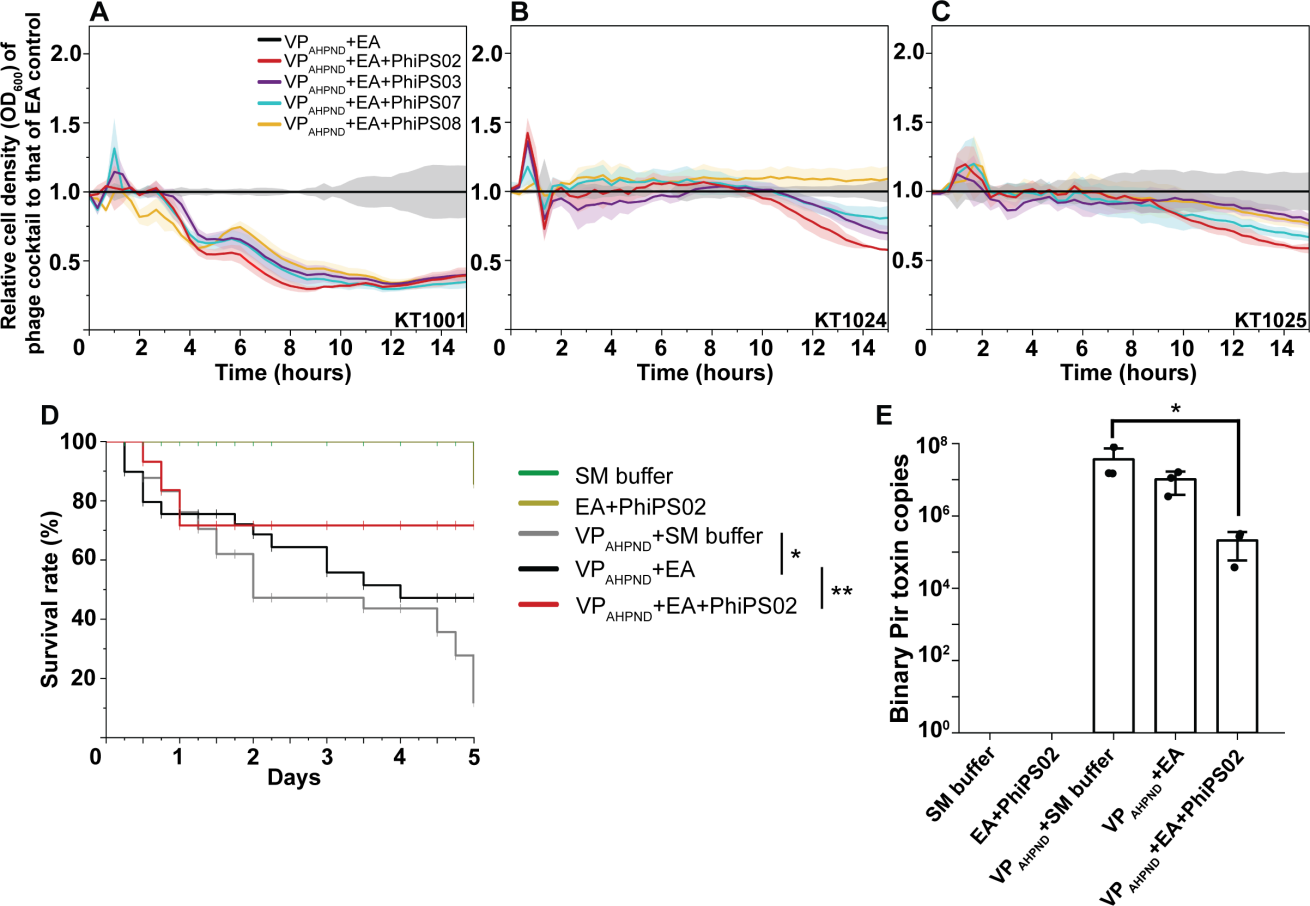
The EA phage cocktail supplemented with PhiPS02 demonstrates potent activity against VP_AHPND_ in both *in vitro* and *in vivo* experiments. *In vitro* bacteriolytic activity of phage cocktails supplemented with candidate phages against VP_AHPND_ strains (**A**) KT1001, (**B**) KT1024, and (**C**) KT1025 at an MOI of 0.01. The graph is represented in relative cell density (OD_600_) of the indicated phage cocktail compared to that of the EA phage cocktail control. (**D, E**) *In vivo* prophylactic experiment in an infection shrimp model, showing (**D**) percentage of shrimp survival rate upon a 5-day bacterial challenge and (**E**) the number of bacterial toxin copies in indicated conditions. *,** indicate statistically significant differences compared with the indicated group (*P* < 0.05 and *P* < 0.01, respectively).

It has long been known that the use of a single phage is insufficient to suppress bacterial growth, whereas a phage cocktail composed of phages exhibiting different infection strategies is more effective than single-phage treatment (10). In our previous study, we discovered nucleus-forming vibriophages EA and demonstrated the potent efficacy of their cocktail (EA cocktail) against various VP_AHPND_ isolates (29). Since we aimed to enhance the efficiency of this existing EA cocktail, we combined each of the selected candidate phages (PhiPS02, PhiPS03, PhiPS07, and PhiPS08) with phage EA as a base formula and then evaluated their enhanced killing activity against deadly VP_AHPND_ isolates (KT1001, KT1024, and KT1025), which can cause 100% shrimp mortality (29). By monitoring bacterial cell density (OD_600_), our results showed that the relative cell density during the treatment of each formula, compared to that of the EA control (Fig. 2A through C; black line), remained at a comparable level during early incubation but then decreased gradually over time in all tested VP_AHPND_ isolates (Fig. 2A through C). These data suggest that the presence of an additional phage in the EA formula enhanced the overall cocktail efficiency compared to the EA cocktail alone. Among the four candidate phages that were included in the formulated cocktails, the supplementation with PhiPS02 (Fig. 2A through C; red line) resulted in the highest reduction of bacterial cell density compared to other combinations, indicating the superior activity of PhiPS02 over other candidates. Additionally, VP_AHPND_ strain KT1001 was more susceptible to this phage cocktail formula, as the relative cell density remained lowest at only 0.39 after 15 hours of treatment, compared to the higher relative cell densities at 0.58 in KT1024 and 0.59 in KT1025.

Given the superior activity of the EA cocktail supplemented with PhiPS02 *in vitro*, we further validated the prophylactic potential of this cocktail formulation *in vivo* (Fig. 2D) using Pacific White Shrimp (*Penaeus vannamei*) as an animal infection model against the most susceptible VP_AHPND_ strain KT1001. We then evaluated the survival rate of shrimp over 5 days under different conditions as follows: (i) SM buffer (control), (ii) EA cocktail supplemented with PhiPS02 (phage cocktail alone), (iii) challenge with VP_AHPND_ without a phage cocktail, (iv) challenge with VP_AHPND_ in the presence of the EA cocktail, and (v) challenge with VP_AHPND_ in the presence of the EA cocktail supplemented with PhiPS02. The result showed that shrimp treated with the phage cocktail alone, without the VP_AHPND_ challenge, survived throughout the 5-day treatment, exhibiting a survival rate comparable to the control group (Fig. 2D; green and yellow lines). This indicates that the phage cocktail is non-toxic and does not cause adverse effects in shrimp. During the VP_AHPND_ challenge, the survival rate rapidly decreased within the first 2 days and continuously declined throughout the 5-day experiment, remaining at only 11% (Fig. 2D; gray line). However, when supplemented with phage cocktails, mortality was delayed, and shrimp were rescued from high death rates. The presence of the EA cocktail (Fig. 2D; black line) increased the survival rate at the end of the experiment to 47%, while supplementation with PhiPS02 (Fig. 2D; red line) maximized the survival rate up to 71%. We further examined Pir toxin copies in the tested animals as an indicator of systemic infection. We found that the VP_AHPND_ challenge led to the highest production of toxin copies at 3.65 × 10^7^ (Fig. 2E). However, treatment with phage cocktails reduced toxin copy numbers, with a significant reduction (approximately 2 log-reduction) observed in the EA cocktail supplemented with PhiPS02 (toxin copies at 2.09 × 10^5^) (Fig. 2E). Altogether, these data suggest that PhiPS02 is the most compatible phage in the EA cocktail, compared to other phage candidates, enhancing the cocktail efficiency by reducing toxin copies produced by VP_AHPND_, which subsequently leads to a higher survival rate in shrimp during infection.

### PhiPS02 is a novel temperate vibriophage

**Fig 3:**
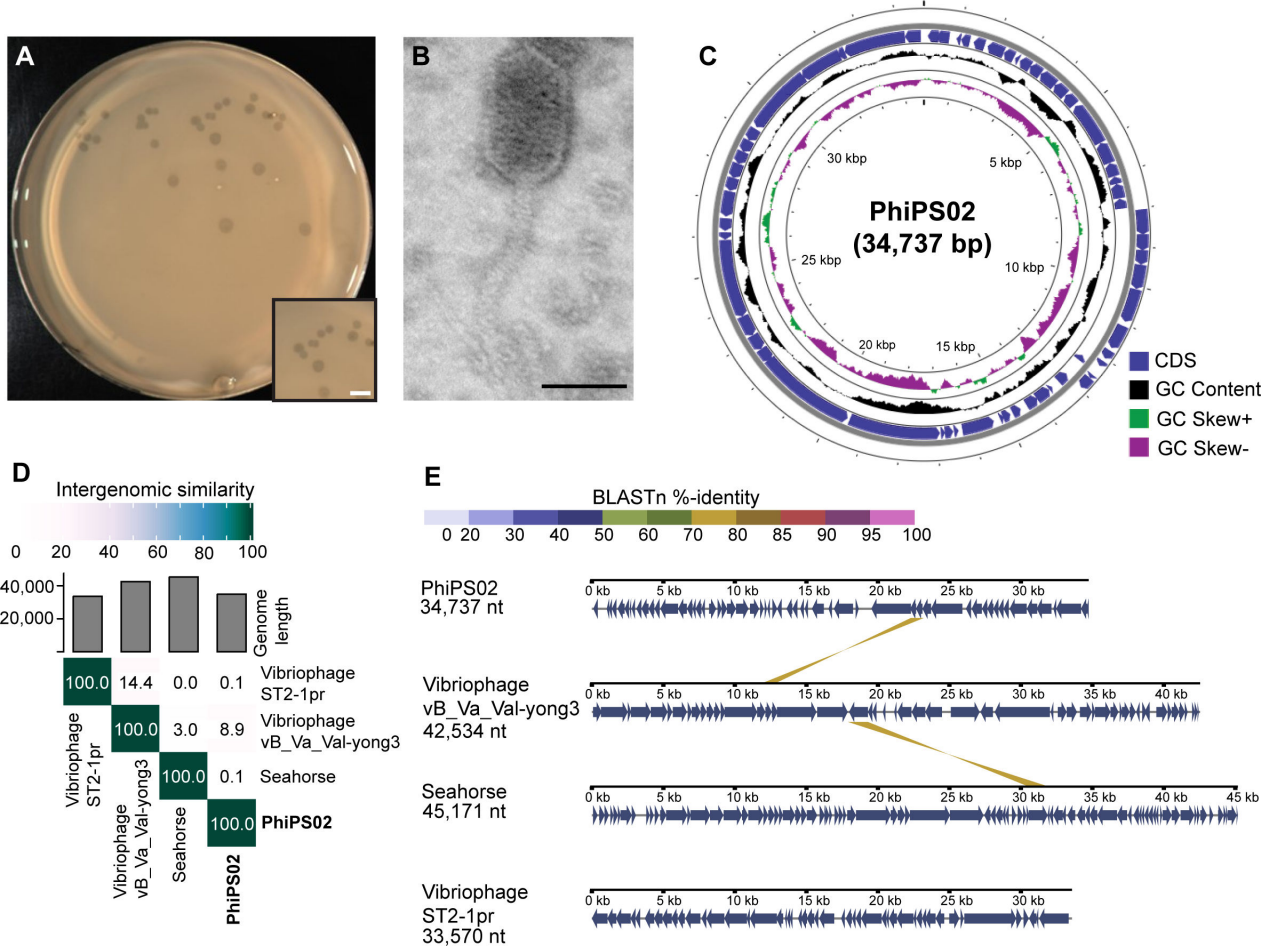
Morphological and biological characteristics of PhiPS02. (**A**) Plaque morphology, scale bars equal to 0.5 cm. (**B**) TEM image at magnification ×40,000, scale bars equal to 50 nm. (**C**) Genome mapping of PhiPS02. PhiPS02 harbors 34,737 base pair genome composed of 61 ORFs. (**D**) Heatmap showing intergenomic similarities of PhiPS02 with other related phages (Vibriophage vB_Va_Val-yong3, Seahorse, and Vibriophage ST2-1pr). The numbers indicated the intergenomic similarity values of each genome pair. (**E**) Comparative genome analysis of PhiPS02, vB_Va_Val-yong3, Seahorse, and ST2-1pr.

Since PhiPS02 is considered a compatible candidate based on its lytic activity that enhances the overall efficacy of the cocktail in both *in vitro* and *in vivo* experiments, we sought to further characterize its genomic and biological properties. PhiPS02 was isolated from water samples collected from seafood trays at a local market in Songkhla province, Thailand, using VP_AHPND_ strain KT1024 as the parental host. The phage formed turbid plaques with the size around 2.03 mm ±0.05 in diameter (*n* = 35) on the bacterial lawn (Fig. 3A). As observed by transmission electron microscopy (TEM) (Fig. 3B), PhiPS02 belongs to the *Siphoviridae* family, characterized by an elongated head (79 nm ±3.46 in length, 44 nm ±2.34 in width; *n* = 5) and a long flexible tail (121 nm ±12.99 in length; *n* = 5).

To further gain deeper insights into its genetic composition, we sequenced the complete whole genome using the Illumina MiSeq platform. The sequencing was successfully assembled into a complete genome (Accession number PV649651.1) with a coverage depth of around 600× (Fig. 3C). Annotation of open reading frames (ORFs) across the phage genomes was conducted using PHASTER and the NCBI database. The analysis showed that PhiPS02 harbors a relatively small genome of 34,737 base pairs, encoding a total of 61 ORFs (Fig. 3C; Table S1). Of those, 24 ORFs were functionally categorized into several groups, including genes involved in DNA replication, transcription and translation, DNA metabolism and modification, virion structure, phage egress, and DNA partitioning. However, 37 ORFs were annotated as hypothetical proteins with unknown functions. Interestingly, PhiPS02 encodes lysogenic-associated genes, including integrase (ORF14) and cI repressor protein (ORF20), along with attachment sites (*att*L, *att*R), which serve as the necessary sequences for prophage genome integration (Table S1). These findings suggest that PhiPS02 is a temperate vibriophage capable of integrating into the host genome.

To evaluate the genetic relationship of PhiPS02 to other known vibriophages, we conducted a Blastn search to first identify closely related phages and then investigated their intergenomic similarity using Virus Intergenomic Distance Calculator (VIRIDIC) analysis. The result demonstrated that, even though vibriophage vB_Va_Val-yong3, vibriophage ST2-1pr, and Seahorse are identified as hits in the NCBI database, PhiPS02 shares extremely low intergenomic similarity to these phages, with similarity indices of 8.9%, 0.1%, and 0.1%, respectively (Fig. 3D). Despite the low overall similarity, PhiPS02 shares a conserved homolog of a minor tail protein with vibriophage vB_Va_Val-yong3, displaying a sequence similarity of 72% (Fig. 3E; Table S1). Taken together, these data suggest that PhiPS02 represents a novel temperate siphophage with the potential to undergo lysogenic conversion.

### Temperate vibriophage PhiPS02 exhibits the ability of lysogenic conversion

**Fig 4:**
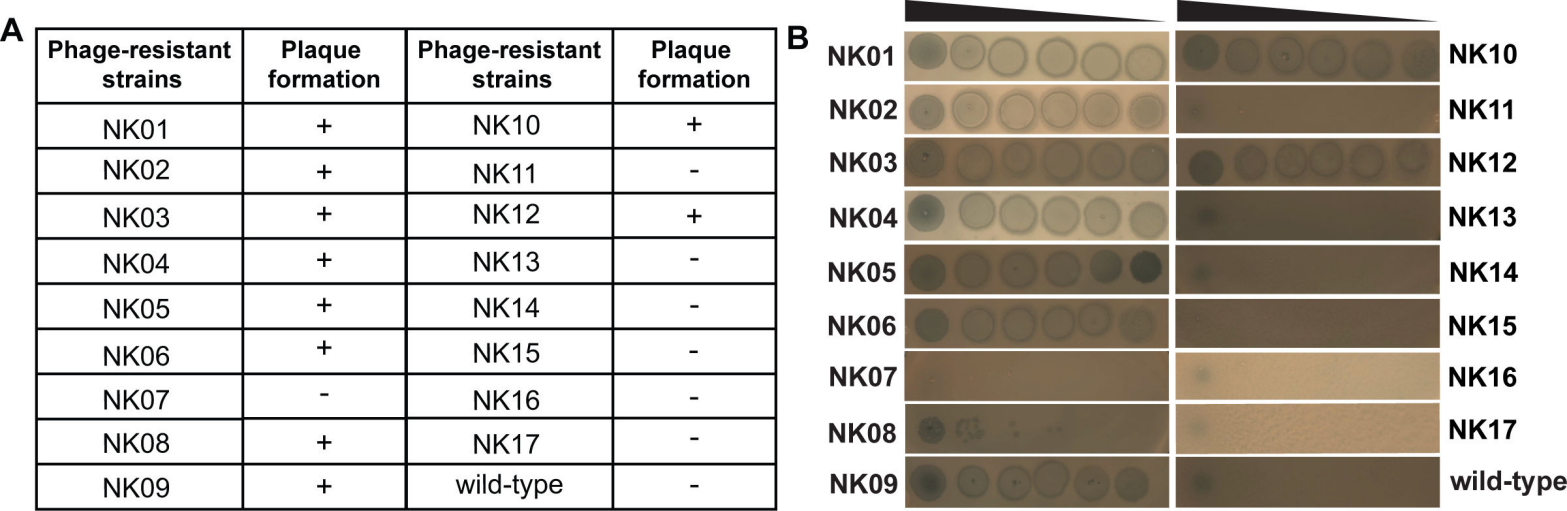
Temperate vibriophage PhiPS02 exhibits the ability of lysogenic conversion. (**A**) MMC treatment for lysogeny induction of 17 phage-resistant strains (NK01-NK17). (+: plaque formation observed and −: no plaque formation observed). (**B**) Serially diluted phage-resistant culture of 17 phage-resistant strains (NK01–NK17) after prophage activation, showing visible plaques, an indicator of the presence of prophage, compared with the wild-type strain.

Temperate phages serve diverse roles in bacterial ecology and viral dynamics, influencing microbial populations through both lytic and lysogenic life cycles (30). The frequency of lysogeny varies among temperate phages due to multiple genetic and environmental factors, further contributing to the complexity of phage-host interplay (31). Bioinformatic analysis of the genome of PhiPS02 identified lysogeny-associated genes, including an integrase (ORF14) and a *cI* repressor protein (ORF20), suggesting its potential for lysogenic conversion. To experimentally confirm this prediction of PhiPS02, we conducted a lysogeny assay. We first raised 47 PhiPS02-resistant bacteria, subsequently screened for stable resistant clones through phage susceptibility testing. Seventeen resistant strains were obtained, and prophage activation was conducted using 2 µg/mL mitomycin C (MMC). The ability of these resistant strains to form plaques was then assessed (Fig. 4A and B). Our result demonstrated that 10 of the 17 PhiPS02-resistant strains (designated NK) are capable of producing clear zones at high phage concentrations (Fig. 4B) and forming visible plaques at lower concentrations (Fig. 4B; NK08), confirming the lysogenic conversion ability of PhiPS02. The plaques also exhibit a bull’s-eye morphology, which is a hallmark characteristic of temperate phages. In contrast, some phage-resistant strains fail to produce plaques, similar to the wild-type strain (Fig. 4B; NK07, NK11, NK13-NK17, and wild type), suggesting that they lack a prophage, and thus their resistance to PhiPS02 is likely mediated through bacterial defenses.

### PhiPS02-lysogenized bacteria produce smaller MVs and a distinct profile of biomolecules compared with wild-type bacteria

**Fig 5:**
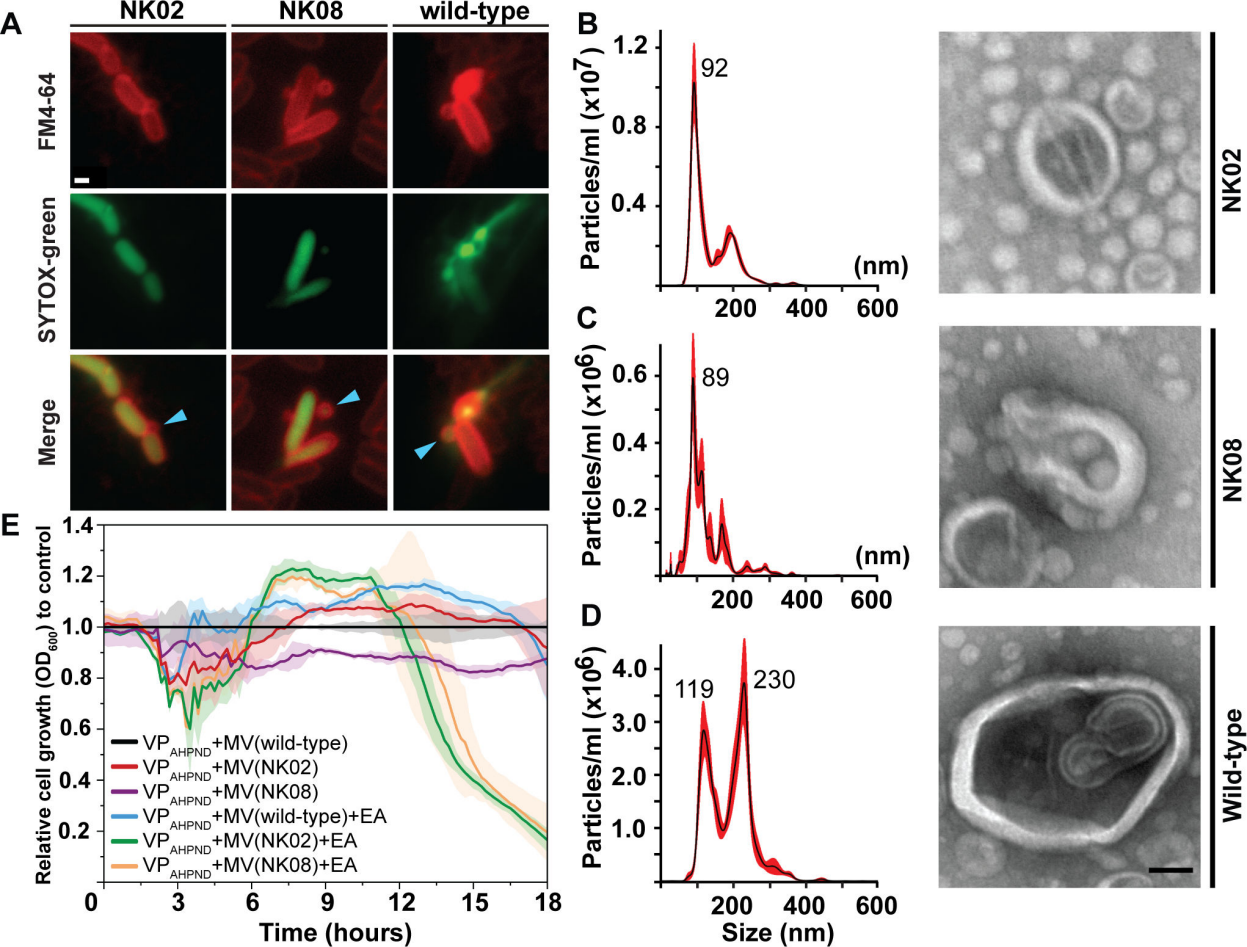
Lysogen-derived MVs enhance the efficiency of the EA phage cocktail. (**A**) Fluorescence images of lysogen strains (NK02 and NK08) and wild-type strain after 4 h of treatment with 0.001 µg/mL MMC. Cell membranes were stained with FM 4-64 dye (red), and permeable cells were positively stained with SYTOX-green (green). Scale bars equal to 1 micron. Blue arrows indicate bacterial MV. (**B–D**, left) Size distribution profiles of bacterial MVs as analyzed by nanoparticle tracking analysis (NTA) and (**B–D**, right) bacterial MVs of lysogen strains (**B**) NK02, (**C**) NK08, (**D**) and wild-type strain, as visualized under TEM. Scale bars equal 50 nm. (**E**) Relative cell growth (OD_600_) of indicated conditions to control (VP_AHPND_ + wild-type derived MV) during an 18-hour period of incubation.

Prophages are known to serve a significant role in enhancing the production of MVs in bacteria (27). While the mechanisms of MV biogenesis differ between Gram-positive and Gram-negative bacteria, some are mediated by phage-encoded enzymes involved in phage egress. These enzymes disrupt bacterial cell walls and membranes, leading to membrane blebbing or cell burst, which subsequently results in MV formation (28, 32, 33). Therefore, we first sought to determine whether the presence of the PhiPS02 prophage in lysogenized strains influences MV production. To induce prophage activity and MV formation, bacterial cultures of representative lysogenized isolates (NK02 and NK08; Fig. 4) were treated with MMC at a concentration (0.001 µg/mL) known to compromise membrane integrity, which potentially leads to cell damage, increased permeability, and subsequent MV release (27). Fluorescence microscopy was used to assess cell integrity, employing SYTOX-Green, an impermeable dye, as an indicator of membrane-compromised cells (Fig. 5A). The result demonstrated that, across all tested strains, NK02, NK08, and wild type, a minor subpopulation (1.5%–3%) exhibits impaired membrane integrity, as evident by the positive staining of SYTOX-Green, regardless of the level of MMC treatment (Fig. 5; Fig. S2). While lysogenized bacteria display a slightly higher proportion of membrane-compromised cells than wild-type bacteria, the difference is not statistically significant, suggesting that the PhiPS02 lysogen does not markedly enhance MV production. Notably, microscopy analysis of SYTOX-Green-stained cells revealed spherical structures that are enclosed within the bacterial membrane, similar to MV-like structures, protruding from the surface of damaged cells (Fig. 5; Fig. S3; blue arrows). These data indicate that both lysogenized and wild-type strains retain the ability for comparable MV formation upon membrane compromise.

To further investigate whether MVs produced by lysogenized and wild-type bacteria differ structurally, we employed nanoparticle tracking analysis (NTA) to determine MV size distribution profiles and TEM to examine MV morphology (Fig. 5B through D). Strikingly, NTA analysis revealed distinct size distribution profiles between MVs produced from lysogenized and wild-type strains. Specifically, the average mode values of the diameter of MVs produced by NK02 (93.6 ± 2.9 nm) and NK08 (90.8 ± 6.5 nm) are approximately 2.2-fold smaller than that produced by wild-type bacteria (205.4 ± 22.3 nm) (Fig. 5B through D, left). TEM imaging data agree well with NTA analysis, assuring the differences in MV particle size among NK02, NK08, and wild-type strains. Moreover, TEM analysis revealed that all MVs produced by bacteria are intact and enclosed within a membrane-like structure, likely derived from the bacterial membrane (Fig. 5A; blue arrows and membrane; red, and Fig. 5B through D, right). Altogether, although the overall rate of MV production does not significantly differ between lysogenized and wild-type bacteria, the presence of the PhiPS02 prophage is associated with MV formation that shifts toward smaller vesicle sizes.

Apart from the difference in MV size, we further evaluated whether the prophage influences the composition and constituents of the MVs. Mass spectrometry data revealed that MVs from both wild-type and lysogenized bacteria contain several receptor and transporter proteins, which can be grouped by functions, including flagellar components and responses, outer membrane proteins, transport, and secretion systems (Table 1). Flagellar-associated proteins and outer membrane protein A (OmpA) were the most abundantly detected in all MVs, suggesting they represent major components on the vesicle surface. Considering the internal constituents of the MVs, the result revealed that MVs produced by lysogenized bacteria (Table 2; NK02 and NK08) harbor several distinct proteins that differ from those of wild-type MVs and are involved in diverse cellular functions such as cell wall and membrane biogenesis, central dogma processes, metabolism, and electron transport. In addition, several phage-derived proteins were identified within the lysogen-derived MVs, confirming that MV biogenesis is triggered by the prophage (Table 2). Altogether, these findings suggest that the PhiPS02 prophage induces the formation of smaller MVs and shapes their unique proteomic profiles that distinguish them from wild-type MVs.

**TABLE 1:** Surface compositions of vesicles derived from wild type, NK02, and NK08

Derived MVs	Protein annotation	Accession number	Functions
wild type	Flagellin	A0A072G9S8	Flagellar components and responses
		A0A072HK07	
		C8CP39	
		A0A1R3F9S1	
		A0A0M0E698	
		A0A072G332	
		A0A072GFE2	
	Flagellin B (fragment)	A0A227JFX9	
	Flagellin C-terminal domain-containing protein (fragment)	A0A227J8Z3	
	Flagellin N-terminal domain-containing protein (fragment)	A0A227J8U9	
	Flagellin hook-associated protein 1	A0A0L7UW49	
	Flagellin hook-associated protein FlgK (fragment)	A0A227J7B3	
	Flagellin hook protein FlgE	Q9X9J8	
		A0A1R3FA18	
	Basal-body rod modification protein FlgD	Q9X9J9	
	OmpA family protein	A0A227JI73	Outer membrane proteins
		A0A072JT35	
		A0A0D8WMX7	
		A0A9Q3UD81	
	OmpA-like domain-containing protein	A0A249W0B4	
		A0AAW3J000	
		Q87PW0	
	Outer membrane beta-barrel protein	A0A7Y0S369	
	Porin family protein	A0A072LLX8	Transport
	ABC transporter permease	A0A249W4W8	
	Acriflavin resistance protein	A0A1R3FCS0	
	Protein translocase subunit SecD	A0A1R3FI69	Secretion systems
	Translocator protein PopB (fragment)	A0A227JFR8	
NK02	Flagellin	A0A072G9S8	Flagellar components and responses
		A0A072HK07	
		C8CP39	
		A0A1R3F9S1	
		A0A072G332	
		A0A0M0E698	
	Flagellin B (fragment)	A0A227JFX9	
	Flagellin C-terminal domain-containing protein (fragment)	A0A227J8Z3	
	Flagellin hook protein FlgE	Q9X9J8	
	OmpA family protein	A0A227JI73	Outer membrane proteins
		A0A0D8WMX7	
	OmpA-like domain-containing protein	A0A249W0B4	
		A0AAW3J000	
	Outer membrane beta-barrel protein	A0AA47JKD6	
	Maltoporin	A0A1R3EFP5	Transport
	Sugar ABC transporter ATP-binding protein	A0A1R3FQ08	
	ABC transporter ATP-binding protein	A0A249WB10	
	Maltodextrin-binding protein	A0A0L8DYD9	
NK08	Flagellin	A0A072HK07	Flagellar components and responses
		A0A0M0E698	
		A0A072G332	
		A0A1R3F9R0	
	Flagellin B (fragment)	A0A227JFX9	
	Flagellin hook protein FlgE	Q9X9J8	
		A0A1R3FA18	
	Chemotaxis protein	A0AA46UQG0	
	Chemotaxis protein CheV (fragment)	A0A7Y0X8V6	
	OmpA family protein	A0A249VZN3	Outer membrane proteins
		A0A0D8WMX7	
	OmpA-like domain-containing protein	A0A249W0B4	
		A0AAW3J000	
	Outer membrane protein	A0A9Q3UAP5	
	Porin	A0A072KZF1	Transport
	Porin family protein	A0A072LLX8	
	Peptide ABC transporter permease	A0A1R3FQC0	
	Ligand-gated channel	A0A249VZ86	
	Type III secretion system translocon subunit VopB	A0AAW8Q7N9	Secretion systems

**TABLE 2:** Constituents of unique proteins within MVs derived from NK02 and NK08 compared to wild type

Function	Protein annotation	Accession number	Mass (Da)	Protein pI	Presented in
Cell wall and cell membrane biogenesis	Conjugal transfer protein	A0AAW3IQB0	12,567	5.01	Both
	Undecaprenyl-phosphate alpha-N-acetylglucosaminyl 1-phosphate transferase	A0A7M1VMX1	40,557	8.99	Both
	Peptidase M23	A0A1R3F9A5	43,017	10.03	NK02
	YeeE/YedE family protein	A0AAW3IV26	14,358	10.21	NK02
	Peptidase S41 (fragment)	A0A227JEI5	53,814	4.89	NK08
	Putative sugar transferase EpsL	A0A7M1VU72	22,828	9.07	NK08
Central dogma	Protein-PII uridylyltransferase	A0A9Q3YJU3	44,815	4.78	Both
	Elongation factor G	A0A1R3FEN5	77,325	4.77	NK02
	Histidine kinase	A0A1R3FS83	192,201	4.77	NK02
	GntR family transcriptional regulator	A0AA46L969	14,519	6.41	NK02
	TetR family transcriptional regulator	A0A1R3F6K4	23,518	6.43	NK02
	ParB/sulfiredoxin domain-containing protein	A0AA46Z5P8	14,232	4.58	NK02
	Transposase	A0A1R3FKB2	59,165	8.83	NK02
	Type I site-specific deoxyribonuclease (fragment)	A0A227JES9	33,474	4.95	NK02
	ATP-dependent RNA helicase RhIE	A0AA46L732	56,362	10.3	NK08
	Exonuclease domain-containing protein	A0AAW8Q7W2	24,868	5.49	NK08
	Large ribosomal subunit protein bL35	A0A072IEJ5	7,384	11.7	NK08
	Ribonuclease	A0A7M1VMI0	51,161	6.68	NK08
	Ribonuclease E	A0A1R3FC45	116,062	9.14	NK08
	XRE family transcriptional regulator	A0AAW3ITK3	73,412	6.56	NK08
Metabolism and electron transport	AAA family ATPase	A0A8H9K4P2	35,075	8.83	NK02
	Alpha/beta hydrolase (fragment)	A0A227J965	24,091	6.45	NK02
	Arginine decarboxylase	A0AAW3J0M4	84,944	5.1	NK02
	ATP-dependent Clp protease ATP-binding subunit ClpA	A0A1R3EV92	83,669	5.46	NK02
	Carboxylate amine ligase	A0AAW3IP74	42,213	8.93	NK02
	Glutathione hydrolase proenzyme	A0A1R3EL10	63,346	5.63	NK02
	Nucleoside hydrolase	A0A1R3F6A5	35,119	4.48	NK02
	PEP-utilizing enzyme mobile domain-containing protein	A0AAX0MFR9	130,077	6.25	NK02
	UbiH protein	Q87LM4	43,658	5.92	NK02
	3-dehydrosphinganine reductase (fragment)	I1SRY0	37,082	7.71	NK02
	3-methyl-2-oxobutanoate hydroxymethyltransferase	A0A1R3FIE3	28,842	5.38	NK02
	Aminotransferase class I/II-fold pyridoxal phosphate-dependent enzyme (fragment)	A0A7Y0SIJ6	9,012	4.58	NK08
	Cytochrome c	A0A1R3F624	11,490	7.74	NK08
	Cytochrome c oxidase assembly protein CtaG	A0A1R3FES8	24,716	6.90	NK08
	Oxygen-dependent choline dehydrogenase	A0A7Y0SQ81	65,065	5.44	NK08
	Phosphoglucosamine mutase	A0A1P8DQ08	11,067	10.90	NK08
Phage-encoded proteins	Phage tail collar domain-containing protein	A0A1R3EDN7	22,773	5.20	NK02
	Putative major tail shaft subunit	PV649651.1 (XUU32949.1)	25,530	4.64	NK02
	Portal protein	A0A0S1WEU8	62,279	5.58	NK08
	Putative portal protein	PV649651.1 (XUU32957.1)	44,508	7.14	NK08
	Hypothetical protein	PV649651.1 (XUU32910.1)	11,289	10.93	NK08
	Hypothetical protein	PV649651.1 (XUU32901.1)	15,663	9.61	NK08
Ungrouped proteins	Uncharacterized protein	A0A1R3EKF4	8,452	8.88	NK02
		A0A9Q3YGI5	20,743	9.19	NK02
		A0AAW3IQC3	10,171	10.06	NK02
		A0A8H9TNL2	59,156	5.42	NK08
		A0AAW3IWZ5	85,798	6.87	NK08
		A0A9E8G723	22,647	8.91	NK08
		A0A9Q3UGM4	12,612	5.66	NK08
		A0A7Z2MSU5	17,064	6.18	NK08
		A0A7M1W4K2	18,504	8.89	NK08
		A0AAW8Q910	19,821	5.48	NK08
		A0AA47JDJ9	43,827	5.83	NK08

### Lysogen-derived MVs enhance the efficiency of the EA phage cocktail

MVs serve diverse roles in phage-host interactions. Recent studies have demonstrated that MVs can facilitate the transfer of bacterial cell surface components and receptors among bacterial populations, thereby resensitizing previously phage-resistant bacteria to phage infection (23). Additionally, MVs contain a variety of biomolecules, the constituents of which vary depending on the bacterial strain of origin. These biomolecules can influence several downstream processes, further shaping the dynamics of phage susceptibility (34, 35). Considering these diverse roles of MVs, along with our findings aforementioned: the distinct structure and compositions of the MVs produced by lysogenized bacteria, and the synergistic activity of the triple-phage combination (EA + PhiPS02), we hypothesized that these MVs specifically induced by PhiPS02 prophage may potentially enhance the bacterial suppression efficacy of the EA phage cocktail. To test this hypothesis, we evaluated the impact of lysogen-derived MV supplementation on EA phage treatment against VP_AHPND_ by monitoring bacterial cell density over an 18-hour period, comparing to the effects of wild-type derived MVs. MVs were extracted and purified from wild-type, NK02, and NK08 strains (Fig. 4), and six formulas were prepared as follows (Fig. 5E): (i) MVs from wild-type bacteria alone, (ii) MVs from NK02 alone, (iii) MVs from NK08 alone, (iv) MVs from wild-type bacteria combined with phages EA, (v) MVs from NK02 combined with phages EA, and (vi) MVs from NK08 combined with phages EA. Bacterial suppression was assessed based on the relative cell growth (OD_600_) of each formula, normalized to the control (wild-type derived MVs alone), to demonstrate the impact of each condition in comparison to wild-type MVs (Fig. 5E). The result revealed that bacterial suppression by phages EA was only substantially enhanced in the presence of lysogen-derived MVs (Fig. 5E; green and orange lines). A reduction in bacterial density was initially observed at approximately 2.5 hours post-incubation (hpi), followed by a slight increase between 5 and 10 hpi. However, the suppression effect becomes more pronounced after 12 hpi (Fig. 5E; green and orange lines). Notably, the bacterial suppression by phages EA in the presence of wild-type MVs (Fig. 5E; blue line) was not as marked as that observed with lysogen-derived MVs. These data suggest that lysogen-derived MVs play a synergistic role in promoting phage infection, thereby leading to more effective bacterial suppression. Specifically, MVs from NK02 and NK08 resulted in a comparable level of reduction in bacterial density at 5.16-fold and 4.34-fold, respectively. However, lysogen-derived MVs alone, in the absence of phages (Fig. 5E; red and purple lines), exhibit only minimal effects on bacterial density, demonstrating that they do not directly suppress bacterial growth themselves but instead rely on the presence of phage to exert their impact.

## DISCUSSION

Since the emergence of VP_AHPND_, including its MDR strains, has caused significant economic losses in the global aquaculture industry, bacteriophages or phages have gained attention as an effective alternative to antibiotics for managing these infections. In this study, we discovered a novel vibriophage, PhiPS02, which enhances the therapeutic potential of our previously developed lytic phage cocktail, composed of vibriophages Eric and Ariel (collectively referred to as EA) (29), against VP_AHPND_ in both *in vitro* and *in vivo* experiments (Fig. 1 and 2). The enhanced antibacterial activities of the newly formulated cocktail in this study (EA + PhiPS02) are likely attributed to the genetic divergence among phages in the combination. Increasing evidence suggests that an effective phage cocktail should include a diverse array of phages as they are likely to recognize different host receptors or employ different mechanisms to hijack bacterial processes (11). The presence of genetically diverse phages allows the cocktail to target multiple critical pathways for bacterial survival simultaneously, thereby making it more difficult for the bacterial host to develop resistance against all phage members at once (11). This concept is supported by an increasing number of studies demonstrating that phage cocktails composed of genetically diverse phages exhibit superior bacterial suppression and more effectively delay the emergence of phage resistance compared to cocktails composed of closely related phages (11, 36). Particularly, vibriophages EA belong to the family Chimalliviridae, a group of nucleus-forming phages that undergo a unique replication strategy (29, 37–39). These chimalliviruses rely on nucleus-based replication, assembling a proteinaceous nucleus-like compartment during the lytic cycle, which is formed primarily by the conserved shell protein Chimallin A (40, 41). This structure facilitates the spatial separation of proteins according to function and protects the phage genome against bacterial defense mechanisms (42, 43). Among the candidate phages tested for compatibility with the EA cocktail, PhiPS02 exhibited the most compatible effect, as evidenced by enhanced bacterial suppression *in vitro* and prophylactic activity in an animal infection model (Fig. 1 and 2). This compatibility is likely due to its pronounced genetic divergence from vibriophages EA (Fig. 3). The PhiPS02 genome is around 34,737 bp, substantially smaller than the genomes of chimalliviruses EA, which are larger than 200 kb. Our genome analysis (Table S1) revealed that, unlike chimalliviruses, PhiPS02 lacks the conserved *Chimallin A* gene, suggesting that it displays a completely different replication strategy. Moreover, while chimalliviruses initiate infection via flagella-dependent adsorption (44), in which the loss of bacterial flagella disrupts the infection and thereby confers resistance, PhiPS02 appears to utilize a different receptor. The inclusion of PhiPS02 in the cocktail was associated with prolonged suppression of bacterial revival (Fig. 2A through C), indicating its potential to infect bacterial cells that have developed resistance to flagella-dependent phages. Further investigation into the infection and replication mechanisms of PhiPS02 will be required to fully understand its contribution to the enhanced efficacy of this genetically diverse phage cocktail.

Due to the low discovery rate of virulent phages against certain critical bacterial pathogens, there has been growing interest and a proposed paradigm shift toward the therapeutic use of temperate phages (12, 15). Recently, temperate phages, also referred to as lysogeny-capable phages, have been employed in combination with lytic phages to treat mycobacterial infections, yielding promising outcomes (15). Studies in *C. difficile* (18) and *Pseudomonas aeruginosa* (17) have further demonstrated that lysogenic conversion mediated by temperate phages can reduce the virulence of their bacterial hosts. Apart from their direct therapeutic applications, temperate phages have also been investigated for their synergistic action with antibiotics. Recent studies in *P. aeruginosa* and *Escherichia coli* have revealed that several antibiotics, particularly those inducing the bacterial SOS response, can influence the lysis-lysogeny balance toward lysis, thereby expanding the potential applications of the temperate phages (45, 46). This expanding application of temperate phages reflects a reconsideration of their potential for therapy under carefully controlled conditions. In this study, the vibriophage PhiPS02 is classified as a temperate phage based on genomic analysis (Fig. 3; Table S1). Specifically, it harbors an integrase gene (*gp014*), which activates the insertion of the viral genome into the host chromosome (30, 47), a *cl* repressor protein (*gp020*) that regulates the lysogenic cycle (48), and attachment sequences (*att*L and *att*R), which serve as attachment sites for prophage integration (49) (Table S1). This genome-based prediction of lysogeny capability (Fig. 3; Table S1) was further supported by experimental validation on PhiPS02 lysogenic conversion (Fig. 4). Given its ability to integrate into the host chromosome, it is important to assess the safety of PhiPS02 to examine the presence of any potential genes or factors that are associated with bacterial virulence in the phage genome (50). Our genome analysis (Fig. S4 and S5) did not identify any antibiotic resistance genes in PhiPS02. However, putative toxin-related genes were predicted, and thus, the sole use of PhiPS02 alone would require cautious monitoring. Apart from concerns mentioned above, the use of temperate phages in cocktails requires careful consideration, as prophage integration can lead to superinfection immunity against other phage infections, which would diminish the effectiveness of other phages in the combination (16). However, our *in vitro* data demonstrate that the EA + PhiPS02 cocktail displays enhanced antibacterial efficacy compared to the EA cocktail alone, as evidenced by significant bacterial suppression (Fig. 2A through C). This observation suggests that superinfection immunity caused by PhiPS02 prophage, if it occurs, does not significantly interfere with the activity of chimalliviruses in the cocktail. Whether PhiPS02 actively mediates superinfection exclusion, or whether chimalliviruses EA possess bypass mechanisms for such immunity, remains elusive and requires further investigation. In addition to *in vitro* findings, the efficacy of the EA + PhiPS02 cocktail tested in an animal infection model demonstrates that this combination also significantly decreased the copy number of VP_AHPND_ toxins to levels below the lethal threshold, leading to increased shrimp survival rates during infection (Fig. 2D and E). Even though these results are promising, further optimization of this triple-phage formulation might be necessary to fine-tune the appropriate phage dosage of maximal bacterial clearance and complete eradication of toxin production to further enhance shrimp survival rates.

The cellular and ecological interplay among temperate phages, lytic phages, and their bacterial hosts within natural communities is inherently complex and dynamic (51). Among many factors influencing phage-host communities, bacterial MVs, which are ubiquitously produced by bacteria and partly induced by phage endolysin-triggered lysis, play a significant role in various physiological processes (33). These include enhancing bacterial survival (52), facilitating biofilm formation (53), mediating antibiotic resistance (54), and, especially, modulating interactions with bacteriophages (19, 33, 55). Since prophage can induce MV biogenesis through endolysin-triggered lysis, these vesicles may indeed influence the outcome of phage combination-based therapies involving temperate phages, which can lead to diverse outcomes, ranging from antagonistic to synergistic interactions. Our results (Fig. 4) showed that all tested vibrio isolates, including the wild-type strain and the lysogens NK02 and NK08, produced MVs at comparable levels (Fig. S2 and S3), regardless of treatment with MMC. However, we observed that the overall frequency of MV release, as indicated by the percentage of permeable cells, was relatively low. This might be attributed to the use of moderate incubation temperature (30°C), which was intended to minimize cellular stress and avoid excessive cell death during the 4-hour MMC exposure period. Despite similar levels of MV production, the size distribution profiles of MVs markedly differed between lysogens and the wild type. Even though all obtained MVs fell within the appropriate size range of spherical outer MVs (OMVs), ranging from 20 to 250 nm in diameter, typically produced by Gram-negative bacteria (53, 56, 57), both lysogens NK02 and NK08 tend to release smaller vesicles (less than 100 nm). This shift in size distribution suggests that the prophage element of PhiPS02 may influence the biogenesis and structural characteristics of vibrio MVs.

The biogenesis of MVs in bacteria is, in part, driven by phage infections, particularly via phage endolysin-triggered explosive cell lysis. In Gram-negative bacteria, an explosive cell burst occurs at the end of the reproduction cycle for phage egress from bacterial cells. This process also introduces a subset of MVs, called E-type MVs, including explosive outer MVs and outer-inner MVs (19, 20). Since MVs are enclosed by the bacterial outer membrane, which harbors various outer membrane proteins, including phage receptors, these MVs can serve several roles during phage and bacterial interactions (19, 20). Bacterial MVs can act as phage decoys by inactivating phages through the adsorption of extracellular phage particles onto exposed receptors on the MV surface, thereby effectively neutralizing the phages and protecting the bacterial population from infection. On the other hand, since MVs carry phage receptors, they can potentially transfer these receptors to phage-resistant isolates via cell-MV fusion, thereby resensitizing them and rendering them susceptible to phage infections (19, 23). Our proteomic data (Table 1) of EV surface compositions revealed the presence of numerous flagellar-related components on all MVs, including flagellin and associated proteins, which serve as receptors for chimalliviruses (44). Even though our data strongly suggested that these MVs do not function as phage decoys as they do not impair phage infection efficiency, they indicate that MVs harboring chimalliviruses receptors may resensitize EA phage-resistant isolates. However, this activity might be influenced by MV size. Our current results (Fig. 5E) demonstrate that only MVs with diameters below 100 nm, derived from lysogens, enhance phage EA activity (Fig. 5E; green and orange lines), whereas larger MVs produced by the wild-type strain do not (Fig. 5E; blue lines). This observation raises the question of whether MV size influences the frequency of cell-MV fusion events and, consequently, the efficiency of phage receptor transfer. Further investigation into the correlation between MV size and receptor transfer frequency will be required.

In addition to the differences in MV size and the presence of phage receptors, the constituents of MVs produced by wild-type and lysogenized bacteria vary significantly, depending on their biogenesis routes (19, 58). In Gram-negative bacteria, MVs, referred to as B-type MVs, are commonly generated through membrane blebbing, while MVs produced by lysogenized bacteria (E-type MVs) are derived from explosive cell lysis. These B- and E-type MVs contain a diverse array of biomolecules, with E-type MVs uniquely harboring components such as phages, phage-derived biomolecules, and other host proteins influenced by phages (19, 20, 59). In our study, the protein profiles of lysogen-derived MVs were distinct from those of wild-type MVs, and the identified proteins can be categorized into multiple functional categories (Table 2). Based on our finding that enhanced bacterial suppression only occurred when supplemented with E-type MVs, we speculate that macromolecules specifically influenced by the PhiPS02 prophage are key contributors to the improved cocktail activity. Among the identified proteins, peptidases detected in both lysogen-derived MVs were of particular interest due to their proteolytic activity, which can compromise cell integrity. In particular, peptidase M23 facilitates phage progeny release during egress and is considered a putative endolysin (60). In contrast, even though peptidase S41 remains poorly understood, it has been implicated in autolysis and cell death (61). The presence of peptidases is commonly observed in MVs from other bacteria (62, 63), where they provide competitive advantages against neighboring bacterial populations through antimicrobial activity (62, 64). Whether the proteolytic activity of these peptidases synergizes with EA phage infection remains to be determined. Collectively, this study highlights the role of prophage-induced MV release in enhancing the efficacy of phage combinations, offering a novel approach to harness temperate phages for therapeutic purposes that warrants further studies.

### Conclusion

We discovered a novel temperate vibriophage, PhiPS02, which enhances the efficiency of our existing EA vibriophage cocktail against VP_AHPND_ infection in both *in vitro* and *in vivo* experiments. This enhancement is achieved through bacterial growth suppression and a reduction in bacterial toxin production, ultimately maximizing shrimp survival. Upon PhiPS02 replication, the phage exhibits the ability of lysogenic conversion, which induces the biogenesis of small bacterial MVs containing distinct biomolecular constituents. Supplementation of these lysogen-derived MVs substantially enhances the bacteriolytic activity of vibriophages EA, suggesting that the improved efficacy of our triple-phage formulation (PhiPS02 + EA) is mediated by MVs produced by lysogenized bacteria.

## MATERIALS AND METHODS

### Bacterial strains and growth conditions

A total of 26 *V*. *parahaemolyticus* strains were used in this study, including 25 AHPND-causing VP strains and 1 non-AHPND-VP strain. All strains were obtained from Songkhla Aquatic Animal Health Research and Development Center. KT1024 and KT1025, which cause high mortality rates in shrimp (29), were used as hosts for phage isolation. All *Vibrio* strains were plated on TSB containing 1.5% agar, and a single colony was inoculated in 5 mL of tryptic soy broth (TSB) supplemented with 2% NaCl (wt/vol) and incubated at 37°C. For experiments that used day culture, overnight culture was inoculated 1:100 into TSB medium until the early exponential growth at optical density (OD_600_ ~ 0.4) at 37°C with shaking at 80 rpm.

### Phage isolation, purification, and propagation

Twelve water samples from the shrimp pond and 10 samples from seafood trays from local markets in Songkhla were used as sources for phage isolation. The phage was isolated using the modified method (65). Briefly, all samples were filtered through a 0.45 µm pore size filter and enriched by mixing with the host (KT1024 and KT1025) at mid-log phase with OD_600_ ~ 0.4 (~10^8^ CFU/mL) with 10× TSB supplemented with 2% NaCl, 0.7% 3-(N-Morpholino) propane sulfonic acid, 4-morpholinepropanesulfonic acid, 1 mM CaCl_2_, and 1 mM MgCl_2_ at final concentration. The mixture was incubated overnight at 37°C with shaking at 100 rpm. After centrifugation at 12,000 rpm for 5 min, the supernatant was filtered through a 0.45 µm pore size filter. Five microliters of each filtrate was dropped on the lawn of VP_AHPND_ strains by the double-layer agar (DLA) method (29). The clear zone was observed, indicating the presence of phages.

For phage purification, the isolated plaque was collected and suspended with 500 µL of SM buffer with gelatin (100 mM NaCl, 8 mM MgCl_2_–7H_2_O, 50 mM Tris-HCl, pH 7.5, 2% gelatin), followed by mixing with VP_AHPND_ strains and using the DLA method. The isolated plaque was purified at least three times to ensure a single virion. For phage propagation, the phage suspension was multiplied with the DLA method using the parental strain as a host. After incubation overnight at 37°C, the top agar was scraped off into a centrifuge tube, and SM buffer (1 M Tris-HCl, pH 7.5, 100 mM NaCl, 8 mM MgCl_2_, and 2% gelatin) was added; the tube was agitated (150 rpm) for 1 h on an orbital shaker and subjected to centrifugation at 8,500 rpm for 15 min. The supernatants were filtered through a 0.45 µm filter. The phage concentration (PFU/mL) was measured using the DLA method and spot tests. The phage lysate was stored at 4°C until use.

### Phage DNA extraction and RFLP

The phage lysate was precipitated by adding phage precipitant solution (10% polyethylene glycol-8000 [wt/vol], 3 M NaCl at final concentration) and mixing gently with the phage lysate. The mixture was stored at 4°C overnight. Then, the mixture was centrifuged at 8,000 rpm for 20 min, followed by discarding the supernatant and leaving the pellet to dry. The pellet was resuspended with SM buffer and treated with DNase I (2 mg/mL) to degrade bacterial genomic DNA, followed by incubation at 37°C for 2 h. The DNase I activity was inhibited by 20 mM EDTA. The phage capsid was digested by lysis buffer (1M Tris, pH 8.0, 0.5 M EDTA, 10% SDS, and 10 mg/mL proteinase K) at 60°C for 1 h. After that, phage DNA was extracted using phenol:chloroform:isoamyl alcohol (25:24:1) extraction. Briefly, an equal volume of phenol:chloroform:isoamyl alcohol was added, mixed, and centrifuged at 12,000 rpm for 5 min. The aqueous layer was transferred and mixed with an equal volume of chloroform and centrifuged at 12,000 rpm for 10 min. The aqueous layer was collected, mixed with 0.3 vol of 3 M sodium acetate and 1 vol of isopropanol, and stored at −20°C overnight. The mixture was centrifuged at 12,000 rpm for 5 min, and the DNA pellet was washed with 70% ethyl alcohol, followed by discarding the supernatant, and the pellet was air-dried, dissolved with nuclease-free water, and stored at 4°C. The DNA concentration was quantified using a NanoDrop spectrophotometer, and the quality of the DNA was assessed by gel electrophoresis.

To determine whether the isolated phages were genetically different, phage genomic DNAs were digested with *Hin*dIII, *Bam*HI, and *Eco*RI restriction enzymes following the manufacturer’s instructions. The digested phage DNAs were separated by gel electrophoresis. Then, clustering the digestion pattern was determined by the unweighted pair-cluster method using the arithmetic averages technique to measure the dissimilarity and construct a phylogenetic tree from a distance matrix.

### Phage host range analysis

The host ranges of the isolated phages were determined against 26 *Vibrio* strains using the DLA method. Briefly, 5 µL of phage lysate was spotted on each lawn of *Vibrio* strains and left to dry at room temperature. The plates were incubated at 37°C overnight. The presence of a clear zone was considered evidence of phage susceptibility. The experiment was performed in independent triplicate.

### Bacterial cell lysis profile

To demonstrate the killing efficiency of the isolated phages against their parental VP_AHPND_ hosts (KT1024 and KT1025), VP_AHPND_ culture at mid-log phase with OD_600_ ~ 0.4 (~10^8^ CFU/mL) was mixed with phage lysate at different MOIs of 0.01, 0.1, 1, and 10, followed by uninfected cells as a control. The mixture was incubated at 37°C. The optical density of bacteria (OD_600_) was measured every 10 minutes until 16 h using a microplate reader. The experiment was performed in triplicate.

### Formulation of a phage cocktail to suppress VP_AHPND_ *in vitro*

To determine phage cocktail activity, four isolated phages (KT1024 host: PhiPS02, PhiPS03; KT1025 host: PhiPS07, and PhiPS08) were selected based on the conventional phage studies and formulated with our previously developed lytic phage cocktail EA that demonstrated the efficacy of EA cocktail against VP_AHPND_ (29). The phage cocktail was formulated by combining EA cocktail with each isolated phage using a ratio of 1:1:1, including (i) PhiPS02: Eric: Ariel, (ii) PhiPS03: Eric: Ariel, (iii) PhiPS07: Eric: Ariel, (iv) PhiPS08: Eric: Ariel, and (v) Eric: Ariel. All phage cocktail formulations were infected with the parental host at an MOI of 0.01, followed by incubation at 37°C. The bacterial growth was measured using a microplate reader every 10 min until 15 h. Cell density (OD_600_) was normalized with VP_AHPND_ and the EA cocktail formula. All treatments were performed in triplicate.

### Potential of the phage cocktail against AHPND-infected shrimp *in vivo*

To validate the prophylactic potential of the cocktail formulation *in vivo*, specific-pathogen-free juvenile *Litopenaeus vannamei* shrimp, each weighing 2–3 g, were provided by the Marine Shrimp Broodstock Research Center II (MSBRC-2), Charoen Pokphand Foods PCL in Phetchaburi Province, Thailand, and were used as an animal infection model against the most susceptible VP_AHPND_ strain KT1001. For supplementation, the basal diet sourced from Charoen Pokphand Foods PCL consists of various ingredients, including squid meal, fish meal, fish oil, soybean meal, wheat flour, wheat gluten, rice bran, fish hydrolysate, squid visceral meal, vitamins, minerals, lecithin, and butylated hydroxytoluene. This commercial diet has the following approximate composition of 38% crude protein, 5% lipid, 11% moisture, and 5% ash.

Two formulations of phage cocktail, PhiPS02 combined with EA phages and EA alone in 10^7^ PFU/mL, were sprayed on basal diets and coated with 1% alpha-starch (wt/wt). The phage cocktail-supplemented diets were air-dried overnight, stored at 4°C, and freshly prepared every 2 days. The control diet consisted of the basal diet supplemented with SM buffer. The experimental shrimp were constantly acclimated in aerated seawater with a salinity of 20 PSU at 28°C ± 1°C for 1 week prior to the experiments.

Healthy shrimps were distributed into three groups, including SM buffer, EA cocktail, and EA cocktail supplemented with PhiPS02. The shrimp were fed twice daily at 3% of the shrimp’s body weight. The leftover diets, molts, and feces were promptly removed. Phage cocktail-supplemented diets were applied to shrimp a day prior to VP_AHPND_ challenge. The fed shrimp were randomly divided into five groups (*n* = 30 each): control groups (SM buffer, EA supplemented with PhiPS02) and VP_AHPND_ challenge groups (SM buffer, EA cocktail, and EA supplemented with PhiPS02 at MOI10). The challenge tanks were exposed to 1 × 10^6^ CFU/mL of VP_AHPND_ by the immersion method. SM buffer with VP_AHPND_ group served as a positive control. Shrimp were continuously fed phage cocktail-supplemented diets throughout the experiment, and cumulative mortality was observed every 6 hours.

The hepatopancreas was collected from three shrimp per group at 5 days post-immersion (dpi) and extracted for gDNA using the Favorprep total genomic DNA extraction kit (Favorgen) to quantify the copy number of VP_AHPND_ binary Pir toxin. A binary Pir toxin region was amplified from extracted gDNA using TUMSAT-VP3 primers (66) via absolute qPCR to assess bacterial load in the experimental shrimp. The reaction was performed using Luna Universal qPCR Master Mix (NEB) with 15 ng of gDNA. The binary Pir toxin copy numbers were calculated using a standard curve of a series of 10-fold dilutions amplified from the DNA plasmid containing binary Pir toxin.

### Transmission electron microscopy

To visualize phage morphology, the negative staining method was used. Ten microliters of phage lysate was spotted on the lawn of the parental host and incubated overnight at 37°C. The clear zone was collected and suspended in 500 µL SM buffer, followed by collection at 4°C overnight. The supernatant was filtered with 0.45 µm filters. The phage was negatively stained with 2% (wt/vol) uranyl acetate (pH 4.5) and placed on grids. Phage morphology was observed by Hitachi HT7700 TEM.

### Phage genome sequencing, genome assembly, and annotation

The phage DNA was extracted using the previous DNA extraction method. DNA quality was evaluated by measuring the absorbance A260/280 ratio, and DNA concentration was measured by a NanoDrop spectrophotometer. Whole-genome sequencing was performed using the Illumina MiSeq. The quality of reads was checked with FASTQ and assembled into contigs with SPAdes. The ORFs were analyzed using the Galaxy server, PHASTER, and annotation using the NCBI server. The genome map was constructed and visualized by the Proksee online service. Intergenomic similarities among related phages were determined by the VIRIDIC. A comparative genome analysis was performed using the DiGAlign online service. Further prediction of the PhiPS02 genome antimicrobial resistance genes using ResFinder version 4.7.2, toxin proteins using CSM-toxin, ToxinPred3.0, DBETH, and virulence factors using VFanalyzer and VirulenceFinder 2.0 web server.

### Phage-resistant *V. parahaemolyticus* screening and lysogeny test

VP_AHPND_ culture at mid-log phase with OD_600_ ~ 0.4 (~ 10^8^ CFU/mL) was mixed with phage lysate at an MOI of 100, followed by incubation for 24 h at 37°C. After incubation, the mixture was diluted with normal saline solution and plated on TSA + 2% NaCl. Then, the plate was incubated overnight at 37°C. The colonies that were able to grow were selected and verified by cell lysis activity and spot test. First, the resistant colonies were purified at least three times to select candidate-resistant strains. Each isolated and wild-type strain was incubated at 37°C for 4 h, and then 100 µL of culture was mixed with 100 µL of phage lysate in 96-well plates. The bacterial growth (OD_600_) profile was measured using a microplate reader every 10 minutes until 16 h of incubation. The experiment was performed in duplicates. The phage-resistant bacteria were determined by a value of OD_600_ that is higher than an infected wild-type strain. These phage-resistant strains were then isolated and validated for their resistance by using the DLA method.

To investigate whether the phage genome can be integrated into the host genome, we induced prophage in isolated phage-resistant strains using MMC, and phage progeny was evaluated by plaque assay. MMC is usually used as a standard agent for phage induction, which induces phages by eliciting bacterial SOS response (67). In brief, a single colony of phage-resistant strains was grown in 500 µL TSB medium then mixed with MMC and incubated at 37°C with shaking at 80 rpm for 4 h. After centrifugation at 9,000 rpm for 3 min, the supernatants were filtered with 0.45 µm filters, and the filtrates were serially diluted followed by a spot test on the lawns of each phage-resistant strain and incubated overnight at 37°C. The clear zone was observed, indicating the phage was a temperate phage with prophage induction.

### Determination of MV formation using fluorescence microscopy

To investigate whether prophages induced MV formation, we compared MV production by using a lysogenized strain harboring a prophage to a wild-type strain. The MMC was used to induce prophage and contribute to MV production (27, 68). To determine the appropriate MMC concentration. The minimum inhibitory concentration (MIC) of MMC, which inhibits host growth, was established in advance. MMC (1 µg/mL) was serially diluted twofold in TSB medium into 96-well plates using the microdilution method. Each of the lysogenized and wild-type strains (10^6^ CFU/mL) was added to the 96 wells containing different MMC concentrations. A positive growth control that contained bacteria without MMC and a negative control of TSB medium only. The culture was incubated at 37°C for 24 h. MIC was determined as the lowest concentration of antibiotic that could inhibit the growth of bacteria. All experiments were performed at least in triplicate.

The MV was produced using MMC induction. Briefly, overnight cultures of the lysogenized and wild-type strain were diluted 100-fold in TSB medium and incubated at 37°C with shaking at 80 rpm until OD_600_ ~ 0.4 (~10^8^ CFU/mL). The MMC (0.001 µg/mL at final concentration) was added into the culture and incubated at 37°C with shaking at 80 rpm for 4 h. After centrifugation at 9,000 rpm for 3 min, the pellets were washed with 1 mL of 1× phosphate-buffered saline (PBS). Then, cell pellets were resuspended in 50 µL of 1× PBS, and 3 µL of the sample was loaded onto an agarose pad (1.2% agarose) that contained 2 µg/mL FM 4-64 for cell membrane staining, and the DNA of permeable cells was stained with 1 µg/mL SYTOX-green for 10 min in the dark at room temperature (27). The samples were visualized under fluorescence microscopy. The resulting images were statistically analyzed and adjusted using the Fiji application.

### Bacterial MVs isolation and characterization

Bacterial MVs were prepared using a previously reported method with modifications (21, 69). In brief, wild-type (KT1024) and lysogenized (NK02, NK08) strains were cultured overnight at 37°C with shaking at 250 rpm in TSB + 2% NaCl. Then, the overnight culture was diluted 100-fold into the fresh TSB + 2% NaCl and cultured at 37°C with shaking at 250 rpm for 17 h. Following this, the culture was centrifuged at 9,000 rpm for 10 min to pellet bacterial cells. The supernatants were filtered with 0.45 µm filters to remove the remaining bacterial cells, and an EDTA-free protease inhibitor cocktail was added to the filtrate. After that, MVs were pelleted by centrifugation at 30,000 rpm for 3 h, 4°C, using a Beckman Coulter Optima L-100XP ultracentrifuge with an SW 41 Ti rotor. After removal of supernatants, MV pellets were resuspended with PBS and stored in aliquots at −80°C.** **

To characterize size distribution profiles and the concentration of MVs measured by NTA using the NanoSight instrument based on Brownian motion of particles in solution. The samples were thawed at room temperature before analysis and were infused in a syringe pump at a speed set as 80 (in arbitrary units). Measurements were captured every 60 s for five rounds, and viscosity was set to 0.89 cP. The images were set up appropriately to enhance the video. The results were analyzed with NTA software version 3.2. Mean size (nm), mode size (nm), and concentration (particles/mL) were calculated and plotted as particle size versus number of particles per mL.

The proteomic composition of MVs was analyzed by mass spectrometry using an ESI-QUAD-TOF instrument and identified by Mascot (version 2.6.0, Matrix Science). MS/MS data were searched against the Uniprot database (*V. parahaemolyticus*) and PhiPS02 database from NCBI (Accession number PV649651.1). Peptides were digested with trypsin/P, and peptide modification was allowed to consider Carbamidomethylation (C, fixed) and Oxidation (M, variable). The results showed a list of peptide-spectrum matches, including score, mass, sequence, and pI value. These peptide identifications are grouped into proteins, showing which proteins are present in the sample, the number of matched peptides per protein, sequence coverage, and overall protein scores.

### Formulation of a phage cocktail and bacterial MVs to suppress VP_AHPND_

To prove whether formulating an EA cocktail with PhiPS02 can enhance the efficacy of the phage cocktail. One reason might be from the effect of MV-producing cells. Partially purified MVs from wild-type, lysogenized strains were used instead of PhiPS02. In the experiment without phage cocktail or MVs treatment, PBS buffer was used as a substitute for both. For the experiment involving phage treatment, the VP_AHPND_ culture (~10^5^ CFU/mL) was combined with the EA cocktail at an MOI of 1. The cocktail mixtures included 6 formulations as follows: (i) VP_AHPND_: MV (wild type), (ii) VP_AHPND_: MV (NK02), (iii) VP_AHPND_: MV (NK08), (iv) VP_AHPND_: MV (wild type): EA, (v) VP_AHPND_: MV (NK02): EA, and (vi) VP_AHPND_: MV (NK08): EA. The bacterial growth was measured using a plate reader every 10 min until 15 h. Cell growth (OD_600_) was normalized to wild-type MVs alone. All treatments were performed in triplicate.

## Data Availability

The genome sequence of phage PhiPS02 has been deposited in NCBI GenBank under accession code PV649651.

## SUPPLEMENTAL MATERIAL

Supplemental material jvi.00941-25-s0001.pdf Figures S1 to S5; Table S1.

ASM does not own the copyrights to Supplemental Material that may be linked to, or accessed through, an article. The authors have granted ASM a non-exclusive, world-wide license to publish the Supplemental Material files. Please contact the corresponding author directly for reuse.
