# Supplementary material for: Enhanced efficiency of virulent and temperate phage combination mediated through bacterial membrane vesicles
Source: J Virol. 2025 Nov 26;99(12):e00941-25. doi: 10.1128/jvi.00941-25 (PMC12724201; doi:10.1128/jvi.00941-25)
Supplement: Supplemental material — Figures S1 to S5; Table S1. [file jvi.00941-25-s0001.pdf]

Enhanced Efficiency of Virulent and Temperate Phage Combination Mediated through Bacterial Membrane Vesicles

Panida Saeju[a,b], Ampapan Naknaen[a,c], Pongsakorn Sukonthamarn[b], Anchalee Tassanakajon[b], Poochit Nonejuie[d], Vorrapon Chaikeeratisak[a,#]

[a] Department of Biochemistry, Faculty of Science, Chulalongkorn University, Bangkok, Thailand

[b] Center of Excellence for Molecular Biology and Genomics of Shrimp, Department of Biochemistry, Faculty of Science, Chulalongkorn University, Bangkok, 10330, Thailand

[c] Department of Biomedical Sciences and Biomedical Engineering, Faculty of Medicine, Prince of Songkla University, Songkhla, Thailand

[d] Center for Advanced Therapeutics, Institute of Molecular Biosciences, Mahidol University, Nakhon Pathom, Thailand

Running Head: Phage synergy via lysogen-derived membrane vesicles

[#]Address correspondence to vorrapon.c@chula.ac.th

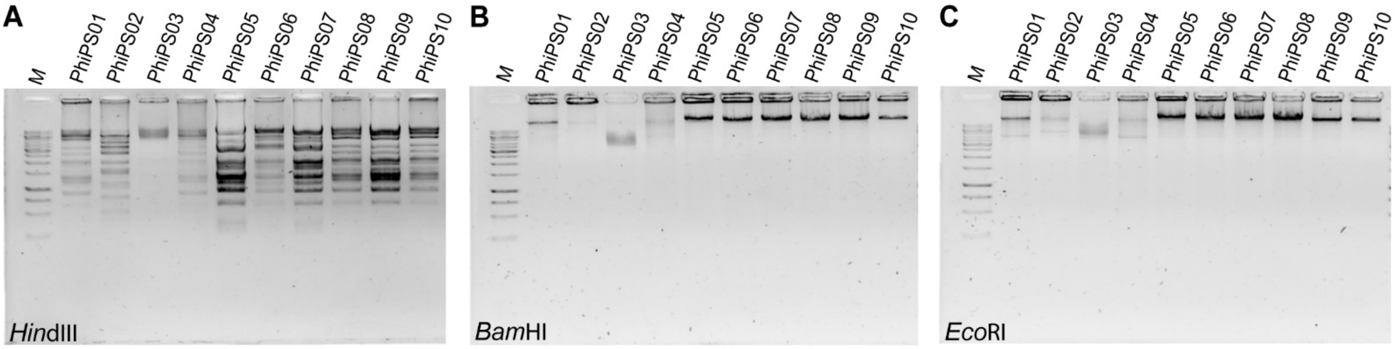

Figure S1. Restriction fragment length polymorphism (RFLP) of the phage genomes digested with the enzyme **(A)** *Hin*dIII, **(B)** *Bam*HI, and **(C)** *Eco*RI. The marker (M) is the 1 kb DNA ladder.

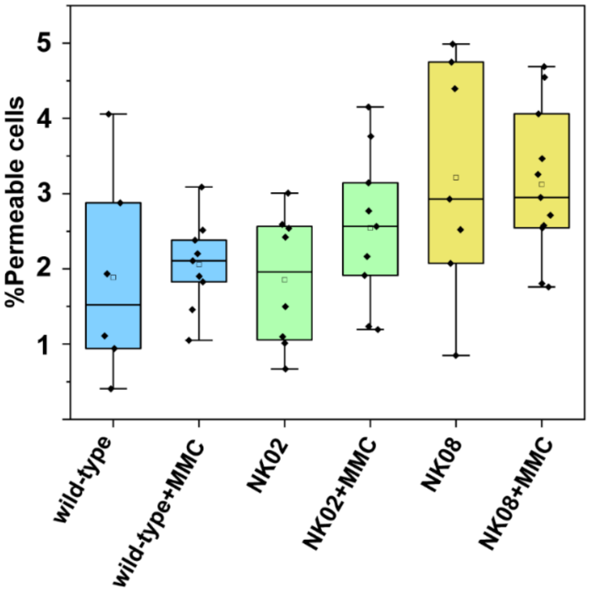

Figure S2. The percentage of permeable cells of lysogen (NK02 and NK08) and wild-type strain with and without 0.001 µg/ml MMC treatment.

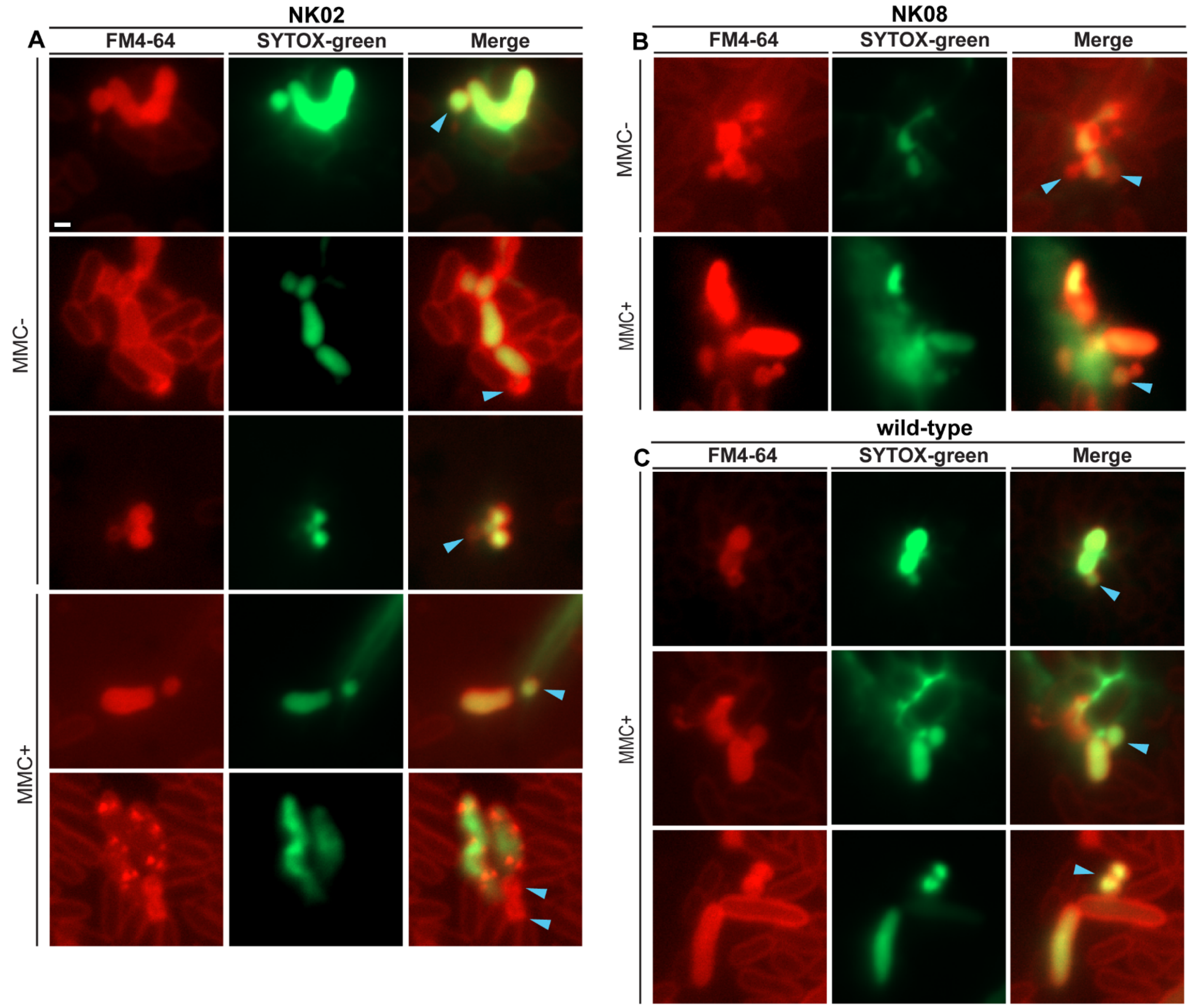

Figure S3. Fluorescence images of lysogen **(A)** NK02, **(B)** NK08, and **(C)** wild-type strains after 4 hour-treatment with or without 0.001 μg/ml MMC (MMC+ or MMC-). Cell membranes were labeled with FM 4-64 dye (red) and permeable cells were stained with SYTOX-green (green). Scale bars equal to 1 micron. Membrane vesicles are indicated by blue arrows.

Antimicrobial class	aminoglycoside																					aminocyclitol	quinolone			beta-lactam				
**Antimicrobial**	amikacin	apramycin	arbekacin	astromicin	butirosin	butiromycin	dibekacin	fortimicin	gentamicin	hygromycin	isepamicin	kanamycin	kasugamycin	lividomycin	neomycin	netilmicin	paromomycin	ribostamycin	sisomicin	streptomycin	tobramycin	spectinomycin	ciprofloxacin	fluoroquinolone	nalidixic acid	amoxicillin	amoxicillin +clavulanic acid	ampicillin	ampicillin +clavulanic acid	aztreonam
**Resistance predicted**	-	-	-	-	-	-	-	-	-	-	-	-	-	-	-	-	-	-	-	-	-	-	-	-	-	-	-	-	-	-

Antimicrobial class	beta-lactam																				*under development	folate pathway antagonist		fosfomycin	glycopeptide			lincosamide	
**Antimicrobial**	cefepime	cefixime	cefotaxime	cefotaxime +clavulanic acid	cefoxitin	ceftazidime	ceftazidime +avibactam	ceftriaxone	cephalothin	cephalotin	ertapenem	imipenem	meropenem	penicillin	piperacillin	piperacillin +clavulanic acid	piperacillin +tazobactam	temocillin	ticarcillin	ticarcillin +clavulanic acid	ceftiofur	sulfamethoxazole	trimethoprim	fosfomycin	bleomycin	teicoplanin	vancomycin	clindamycin	lincomycin
**Resistance predicted**	-	-	-	-	-	-	-	-	-	-	-	-	-	-	-	-	-	-	-	-	-	-	-	-	-	-	-	-	-

Antimicrobial class	streptogramin A				pleuromutilin	macrolide							tetracycline				streptogramin B			oxazolidinone	amphenicol		polymyxin	steroid antibacterial	pseudomonic a^	rifamycin	nitroimidazole	ionophores		
**Antimicrobial**	dalfopristin	pristinamycin IIA	quinupristin +dalfopristin	virginiamycin M	tiamulin	azithromycin	carbomycin	erythromycin	oleandomycin	spiramycin	telithromycin	tylosin	doxycycline	minocycline	tetracycline	tigecycline	pristinamycin IA	quinupristin	virginiamycin S	linezolid	chloramphenicol	florfenicol	colistin	fusidic acid	metronidazole	mupriocin	rifampicin	narasin	maduramicin	salinomycin
**Resistance predicted**	-	-	-	-	-	-	-	-	-	-	-	-	-	-	-	-	-	-	-	-	-	-	-	-	-	-	-	-	-	-

- : No resistance and no match found in database

Figure S4. Prediction of antimicrobial resistance genes of the PhiPS02 genome using ResFinder version 4.7.2. The hyphen (-) indicates no resistance and no match found in the database.

A

Server		ORF1	ORF2	ORF3	ORF4	ORF5	ORF6	ORF7	ORF8	ORF9	ORF10	ORF11	ORF12	ORF13	ORF14	ORF15	ORF16	ORF17	ORF18	ORF19	ORF20	ORF21	ORF22	ORF23	ORF24	ORF25	ORF26	ORF27	ORF28	ORF29	ORF30
**CSM-toxin**	**probability**	0	0	0	0	.15	0	0	0	.04	0	0	0	0	0	0	0	0	0	.02	0	0	0	0	0	0	0	0	0	0	0
	**prediction**	–	–	–	–	–	–	–	–	–	–	–	–	–	–	–	–	–	–	–	–	–	–	–	–	–	–	–	–	–	–
**ToxinPred 3.0**	**probability**	0	0	.54	0	0	0	0	0	0	0	0	.06	0	0	0	0	0	0	0	0	0	0	0	0	0	0	0	0	0	0
	**prediction**	–	–	+	–	–	–	–	–	–	–	–	–	–	–	–	–	–	–	–	–	–	–	–	–	–	–	–	–	–	–

Server		ORF31	ORF32	ORF33	ORF34	ORF35	ORF36	ORF37	ORF38	ORF39	ORF40	ORF41	ORF42	ORF43	ORF44	ORF45	ORF46	ORF47	ORF48	ORF49	ORF50	ORF51	ORF52	ORF53	ORF54	ORF55	ORF56	ORF57	ORF58	ORF59	ORF60	ORF61
**CSM-toxin**	**probability**	0	0	0	0	0	0	0	0	0	0	0	0	0	0	0	0	.20	0	0	0	0	0	0	0	0	0	0	1	0	0	0
	**prediction**	–	–	–	–	–	–	–	–	–	–	–	–	–	–	–	–	–	–	–	–	–	–	–	–	–	–	–	+	–	–	–
**ToxinPred 3.0**	**probability**	0	0	0	0	0	0	0	0	0	.31	.07	0	0	0	0	0	0	0	0	0	0	0	.02	0	0	0	0	0	0	.18	0
	**prediction**	–	–	–	–	–	–	–	–	–	–	–	–	–	–	–	–	–	–	–	–	–	–	–	–	–	–	–	–	–	–	–

– : Non-toxin (probability<0.5)
+ : toxin (probability≥0.5)

	Search	Prediction
**DBETH server**	Toxin sequences	low-confidence hit, low query coverage and non-significant E-value<br>***The sequence is not strongly predicted to be a toxin.***
	Toxin domains from<br>-CDD database<br>-SMART database	 No hit found<br>No hit found

DBETH: Database of Bacterial ExoToxins for Humans

B

	Web-server	Prediction
**Virulence factor (VF) prediction**	**VFanalyzer** (VFDB)	No VF found
	**VirulenceFinder 2.0**<br>-organism: *E. coli*<br>-organism: *S. aureus*	 No hit found<br>No hit found

Figure S5. (A) Toxin proteins predicted from the PhiPS02 genome using CSM-toxin, ToxinPred3.0, and DBETH web servers. CSM-toxin and ToxinPred3.0 show the probability for ORFs as a toxin in the upper panel and indicate the prediction result in the lower panel (+; toxin or -; non-toxin). **(B)** Virulence factor prediction using VFanalyzer from the virulence factor database (VFDB) and VirulenceFinder 2.0 web servers.

Table S1 List of annotated proteins from ORFs in PhiPS02 genome including bacterial attachment sites.

ORF	Predicted function	Direction	Start	Stop	Size (nt)	Size (aa)	Sequence similarity	E-value
	*att*L		1	77	77			
1	Hypothetical protein	-	104	406	303	100		
2	HNH endonuclease	-	349	708	360	123	PHAGE_Entero_phiP27_NC_003356: hypothetical protein	3.2E-48
3	Hypothetical protein	-	907	1029	123	40		
4	Hypothetical protein	-	1038	1286	249	82		
5	Hypothetical protein	-	1395	1715	321	106		
6	Hypothetical protein	-	1798	2283	486	161	PHAGE_Altero_vB_AmaP_AD45_P1_NC_021532: hypothetical protein	4.38E-25
7	Holin	-	2289	2591	303	100	PHAGE_Burkho_BcepSaruman_NC_049850: Holin	2.84E-14
8	Hypothetical protein	-	2635	2745	111	36		
9	Hypothetical protein	-	2784	3116	333	110		
10	Transcriptional regulator	-	3113	3463	351	116	PHAGE_Marino_P12026_NC_018269: hypothetical protein	5.32E-12
11	Hypothetical protein	-	3469	3963	495	164	PHAGE_Mycoba_SirDuracell_NC_041987: hypothetical protein	1.94E-17
12	Hypothetical protein	-	3966	4364	399	132	PHAGE_Burkho_phi1026b_NC_005284: gp45	2.06E-07
13	Hypothetical protein	-	4377	4739	363	120		
14	Integrase	-	4789	6027	1239	412	PHAGE_Vibrio_X29_NC_024369: integrase	6.18E-79
15	Exonuclease	-	6032	6643	612	203	PHAGE_Vibrio_X29_NC_024369: exonuclease	6.35E-38
16	Hypothetical protein	-	6650	7036	387	128		
17	Hypothetical protein	-	7047	7253	207	68		
18	Hypothetical protein	-	7253	7663	411	136		
19	Hypothetical protein	-	7675	7956	282	93		
20	*cI* repressor protein	+	8196	8684	642	213	PHAGE_Pseudo_PMG1_NC_016765: *cI* repressor protein	2.76E-06
21	Hypothetical protein	+	8674	9081	408	135		
22	Hypothetical protein	+	9071	9442	372	123		
23	DNA methyltransferase	+	9445	10068	624	207	PHAGE_Cronob_vB_CsaP_009_NC_048664: DNA methyltransferase	6.48E-28
24	Hypothetical protein	+	10078	10944	867	288		
25	ParA-like protein	+	11059	11688	630	209	PHAGE_Sodali_phiSG1_NC_007902: ParA-like protein	9.06E-10
26	Hypothetical protein	+	11764	12003	240	79		
27	Hypothetical protein	+	12084	12233	150	49		
28	Hypothetical protein	-	12276	12443	168	55		
29	Hypothetical protein	+	12518	12622	105	34		
30	DNA-binding protein	+	12626	12952	327	108	PHAGE_Vibrio_vB_VpaM_MAR_NC_019722: DNA-binding protein	2.93E-29
31	Hypothetical protein	-	12956	13267	312	103	PHAGE_Burkho_KS14_NC_015273: gp6	1.04E-08
32	Hypothetical protein	-	13506	13598	93	30		
33	Hypothetical protein	-	13612	14037	426	141	PHAGE_Vibrio_1.202.O.10N.222.45.E8_NC_048066: coil containing protein	6.03E-31
34	Hypothetical protein	-	14030	14374	345	114		
35	Hypothetical protein	-	14571	14816	246	81		
36	Hypothetical protein	-	14887	15111	225	74		
37	Hypothetical protein	-	15089	15205	117	38		
38	Hypothetical protein	-	15389	16297	909	302		
39	Hypothetical protein	-	16402	16530	129	42		
40	Hypothetical protein	-	16553	16786	234	77		
41	Hypothetical protein	-	16776	16901	126	41		
42	Hypothetical protein	-	16925	19483	2559	852		
43	Tail protein	-	19547	22306	2760	919	PHAGE_Pseudo_PS_1_NC_029066: tail protein	4.97E-44
44	Hypothetical protein	-	22263	22670	408	135		

45	Minor tail protein	-	22667	23119	453	150	PHAGE_Pseudo_PMG1_NC_016765 : minor tail protein	4.81E-15
46	Putative minor tail protein	-	23122	23715	594	197	PHAGE_Vibrio_ vB_Va_Val-yong3: minor tail protein	5.00E-88
47	Putative tail tape measure protein	-	23715	25907	2193	730	PHAGE_Vibrio_VpKK5_NC_026610: putative tail tape measure protein	3.99E-85
48	Hypothetical protein	-	25918	26118	201	66		
49	Hypothetical protein	-	26160	26510	351	116		
50	Putative Major tail shaft subunit	-	26559	27269	711	236	PHAGE_Klebsi_phiKO2_NC_005857: major tail shaft subunit	1.76E-17
51	Tail terminator	-	27285	27656	372	123	PHAGE_Klebsi_phiKO2_NC_005857: Gp10	2.99E-05
52	Hypothetical protein	-	27653	28078	426	141	PHAGE_Klebsi_phiKO2_NC_005857: Gp11	3.34E-26
53	Putative head-tail adaptor protein	-	28075	28434	360	119	PHAGE_Paenib_Harrison_NC_02874 6: head-tail adaptor protein	3.62E-05
54	Putative head-tail connector protein	-	28431	28784	354	117	PHAGE_Shigel_SfII_NC_021857: head-tail connector protein	1.55E-19
55	Hypothetical protein	-	28788	29153	366	121		
56	Major capsid protein	-	29213	30553	1341	446	Minor capsid protein [Streptococcus phage Javan93]	3.00E-78
57	Head maturation protease	-	30417	31139	723	240	PHAGE_Entero_c_1_NC_019706: head maturation protease	7.57E-58
58	Putative portal protein	-	31111	32316	1206	401	PHAGE_Burkho_phiE125_NC_00330 9: putative portal protein	6.00E-68
59	Hypothetical protein	-	32273	32476	204	67		
60	Terminase large subunit	-	32473	34206	1734	577	PHAGE_Psychr_Psymv2_NC_02373 4: terminase large subunit	0.0
61	Terminase small subunit	-	34206	34649	444	147	PHAGE_Klebsi_phiKO2_NC_005857: putative small terminase subunit	5.35E-18
	*att*R	-	34661	34737	77			
